# Tracing the history and ecological context of *Wolbachia* double infection in a specialist host (*Urophora cardui*)—parasitoid (*Eurytoma serratulae*) system

**DOI:** 10.1002/ece3.2713

**Published:** 2017-01-17

**Authors:** Jes Johannesen

**Affiliations:** ^1^ Department of Evolutionary EcologyZoological InstituteUniversity of MainzMainzGermany

**Keywords:** community analysis, horizontal transmission, immigration, parapatry, pupae developmental stage

## Abstract

The endosymbiotic bacterium *Wolbachia* is the most widespread bacteria in insects, yet the ecology of novel acquisitions in natural host populations is poorly understood. Using temporal data separated by 12 years, I tested the hypothesis that immigration of a parasitoid wasp led to transmission of its *Wolbachia* strain to its dipteran host, resulting in double‐strain infection, and I used geographic and community surveys to explore the history of transmission in fly and parasitoid. Double infection in the fly host was present before immigration of the parasitoid. Equal prevalence of double infection in males and females, constant prevalence before and after immigration in two regions, and increase in one region of immigration indicate little if no competition between strains. Double infection was present throughout the fly's distribution range, but proportions varied highly (0–0.71, mean = 0.26). Two fly‐specific MLST strains, observed in Eastern and Western Europe, respectively, differed at hcpA only. Flies with either fly‐strain could be double infected with the parasitoid's strain. The geographic distribution of double infection implies that it is older than the fly host's extent distribution range and that different proportions of double infection are caused by demographic fluctuations in the fly. The geographic data in combination with community surveys of infections and strains further suggest that the parasitoid strain was the fly's ancestral strain that was transmitted to the parasitoid, that is, the reverse transmission route as first hypothesized. Based on these findings together with a comparison of oviposition strategies of other hosts harboring related *Wolbachia* strains, I hypothesize that trans‐infection during an insect host's puparial metamorphosis might be important in promoting horizontal transmission among diverse holometabolic taxa.

## 1 Introduction

The maternally inherited intracellular bacterium *Wolbachia pipientis* Hertig 1936 is likely the most widely distributed endosymbiont in insects (Ahmed, Araujo‐Jnr, Welch, & Kawahara, 2015a; Hilgenboecker, Hammerstein, Schlattmann, Telschow, & Werren, 2008). This facultative endosymbiont is estimated to be present in about 40% of all insect species (Zug & Hammerstein, 2012). Facultative associations are prone to turnover, and this may channel interspecific exchange. For *Wolbachia* specifically, incongruent cophylogenies with hosts (Shoemaker et al., 2002; Vavre, Fleury, Lepetit, Fouillet, & Bouletreau, 1999), the observation that phylogenetically diverse taxa share identical or similar *Wolbachia* strains (Huigens, de Almeida, Boons, Luck, & Stouthamer, 2004; Noda et al., 2001) and recombination between strains (Bordenstein, Wernegreen, & Werren, 2006; Werren & Bartos, 2001) indicate that interspecific transmissions of *Wolbachia* are common on an evolutionary time scale (Ahmed, Breinholt, & Kawahara, 2016; Baldo et al., 2008; O'Neill, Giordano, Colbert, Karr, & Robertson, 1992; Russell, Latorre, Sabater‐Muñoz, Moya, & Moran, 2003). Yet the number of studies documenting interspecific transmission or spread of strains in natural populations remains few (e.g., Hoshizaki & Shimada, 1995; Kriesner, Hoffmann, Lee, Turelli, & Weeks, 2013; Schuler et al., 2013; Turelli & Hoffmann, 1991).

The circumstances involved in interspecific transmission in natural populations are mostly enigmatic, but agents such as shared resources (Ahmed et al., 2016; Schuler et al., 2013), hybridization (Raychoudhury, Baldo, Oliveira, & Werren, 2009; Whitworth, Dawson, Magalon, & Baudry, 2007) and predator–prey interactions (Werren & Bartos, 2001) have been shown to stand in relation to acquisition. While the direction of transmission in predator–prey systems may occur in both directions (Hughes, Pamilo, & Kathirithamby, 2004), transmission via the ovipositor of a parasitoid (“dirty needle” hypothesis, sensu Houck, Clark, Peterson, & Kidwell, 1991) has been given recent attention. Ahmed et al. (2015b) elegantly showed how nonlethal inspection of whitefly nymphs by a parasitoid wasp could transfer *Wolbachia*. The transmission was phoretic, that is, mechanical, and although the infection was lost after about 5 days, observed fitness benefits to infected whitefly populations as well as the generalist nature of the parasitoid allow for the possibility of transfer among and hereditary establishment in novel species. Genty, Bouchon, Raimond, and Bertaux (2014) suggested that *Wolbachia* infection of the soma might serve as a reservoir for germline infection.

Environmental change has further been stressed as an important, albeit indirect agent because it has the potential to create new biotic interaction levels. Rocha, Mascarenhas, Perondini, and Selivon (2005) reported acquisition of *Wolbachia* in invasive Brazilian populations of the fly *Ceratitis capitata*. Schuler et al. (2013) surveyed the acquisition of a strain infecting European cherry fruit fly, *Rhagoletis cerasi*, in invasive Eastern American cherry fruit fly, *Rhagoletis cingulata*, in Europe. Both species depend on cherries for oviposition and larval development. This suggests transmission via their common food/oviposition resource, but signs of hybridization between the two species have also been reported (Johannesen, Keyghhobadi, Stauffer, Schuler, & Vogt, 2013). Alternatively to transmission, Reuter, Pedersen, and Keller (2005) reported loss of *Wolbachia* in an invasive ant species.

**Figure 1:**
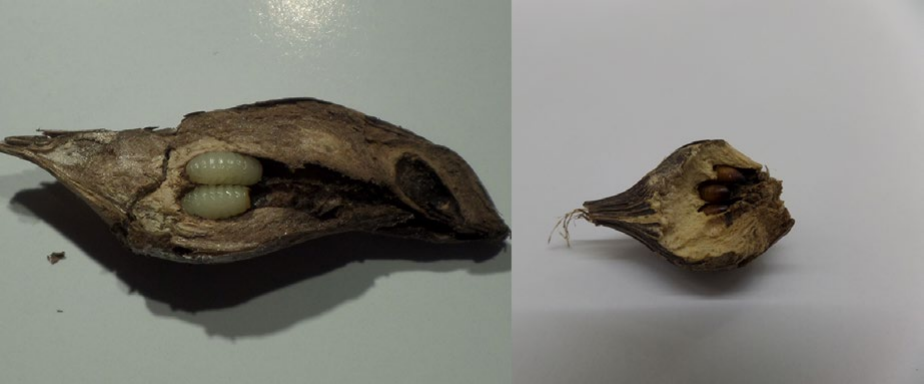
Dissected *Urophora cardui* galls showing nonparasitized *U. cardui* larvae (left) and pupae parasitized with *Eurytoma serratulae* (right)

In this study, I address *Wolbachia* infection in an ecological context by exploring the evidence for transmission between the specialist endoparasitoid, *Eurytoma serratulae* Latr. (Eurytomidae) and its fly host *Urophora cardui* L (Tephritidae) from natural populations. The fly is a specialized gall maker on creeping thistle *Cirsium arvense* (L.) (Scop) (Asteraceae). The specialist endoparasitoid *E. serratulae* and the generalist ectoparasitoid *E. robusta* Mayr are the main parasitoids. Both are found throughout the distribution range of the host in Europe to the Ural Mountains (Frenzel, Eber, Klotz, & Brandl, 2000; Zwölfer, Böheim, & Beck, 2007) with one known exception, the Jutland (Cimbrian) peninsula where *E. serratulae* was absent until 2001 (Johannesen & Seitz, 2003a). The peninsula, situated between the North and Baltic Seas and extending northwards from Hamburg, Germany, into Denmark, constitutes an area of about 50,000 km^2^. Surveys from 1999 to 2006 recorded progressive northward expansion of *E. serratulae* there: none in 1999, single individuals in the south in 2001, and in 2006 in the center of the peninsula. Surveys between 2013 and 2015 showed that *E. serratulae* is now firmly established (Johannesen & Seitz, 2003a; Johannesen & Prill, this study). *E. serratulae* attacks early second stage *U. cardui* larvae at the beginning of gall formation (Basov, 2002; Schlumprecht, 1989). *Eurytoma serratulae* larvae stay in developmental stasis until late summer when they induce early pupation and consume the fly larvae (Claridge, 1961) before diapausing singly as a final stage larva within a puparia. Nonparasitized fly larvae, by contrast, diapause as larvae and pupate shortly before emergence in spring (Figure 1). The area of *E. serratulae* immigration harbors genetically distinct *U. cardui* populations, which are separated by a transition zone across the center of the peninsula (Johannesen, Drüeke, & Seitz, 2010; Steinmetz, Johannesen, & Seitz, 2004).

While testing *U. cardui* for *Wolbachia* for being a putative agent influencing the transition zone, I observed that *U. cardui* north of the zone were double infected with a *Wolbachia* strain otherwise associated with *E. serratulae*, whereas *U. cardui* in *E. serratulae*'s ancestral area just south of the transition zone were not. Based on these findings, I speculated that *U. cardui* in the north had become infected with the parasitoid strain after immigration of the parasitoid and that the acquisition of double infection (DI) might impact the separation of *U. cardui* on either side of the transition zone (Riegler & Stauffer, 2002; Werren, 1998). Confirmation of the hypothesis would represent evidence for recent horizontal transmission between interacting species and highlight how environmental change (i.e., parasitoid immigration) might influence both the spread and acquisition of new infections as well as promote genetic diversification between host populations. To test the hypothesis that DI in *U. cardui* is embedded in an interaction with *E. serratulae*, I employed a three‐level analysis. First, to specifically test the novelty of DI in the immigration area, I assayed *Wolbachia* infection in individuals sampled before (2001) and after (2013–2014) colonization by *E. serratulae*. Horizontal transmission from *E. serratulae* to *U. cardui* is supported when DI in *U. cardui* in the immigration area was absent in 2001 but widespread in 2014, and absent in the ancestral range of co‐occurrence with *E. serratulae*. Second, I surveyed infections across the distribution range of *U. cardui* and *E. serratulae*. This survey gave insights into whether the presence and/or absence of DI are unique to the immigration area but also assessed the age and stability of DI. Third, I studied *Wolbachia* infection at the community level to evaluate the alternative hypotheses that (1) *Urophora cardui* was the original host of the parasitoid strain and/or (2) DI was related to other interacting species immediately associated with *U. cardui*'s life cycle. Together, the three levels infer the history of DI in *U. cardui*.

## 2 Materials and Methods

### 2.1 Handling and geographic sampling

All investigated species were collected and reared in an identical way. All species hibernate as end‐stage larvae in lignified galls. Galls were collected between end‐October to mid‐November of a given year, stored at 3–7°C for 5 months after which they were opened and the larvae removed. Larvae were raised singly to imago in 1.5 ml Eppendorf tubes perforated with two holes. Upon emergence, all individuals were stored at −20°C until analysis. Hence, all adult insects were virgin and without contact to other individuals during end‐metamorphosis (pupation) or as adults.

**Figure 2:**
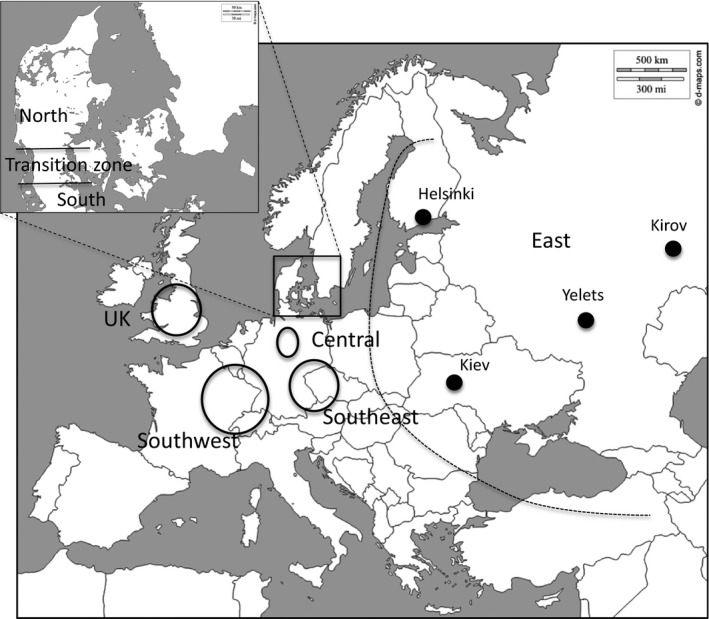
Map of Europe showing sampling regions defined by genetic differentiation in the fly *Urophora cardui*. The parasitoid *Eurytoma serratulae* was absent on the Jutland peninsula (insert) before 2002 but present elsewhere. Fly populations in Jutland are divided by a genetic transition zone that separates a divergent population in North Jutland (North) from other regional populations. The specific sample locations and sample sizes per location and region are shown in Table 1

Analysis of *Wolbachia* infection in *U. cardui* was based on individuals sampled in 2001–2003 and 2013–2014 (Table 1). Analysis was performed near exclusively on adults, *N* = 508 adults + 12 larvae = 520 specimens, with the purpose of avoiding the potential bias of parasitoid encapsulation in larvae that might impact estimates of infection. The frequency of single infections (SI) and double infections was compared among regional populations previously identified (Johannesen et al., 2010; Steinmetz et al., 2004) (Figure 2). The regions represent populations north, within and south of a genetic transition zone on the Jutland peninsula. Regions for comparisons were southeast (southeast Germany, Austria), southwest (southwest Germany, France), central Germany, and east (Russia, Ukraine, and Finland) and Great Britain (England). *Wolbachia* infection in *E. serratulae* and the generalist ectoparasitoid *E. robusta* were screened throughout the distribution area of *U. cardui* from the same locations and years as *U. cardui*. Infection of the *U. cardui*‐associated parasitoids, *Torymus chloromerus* Walker and *Pteromalus elevatus* Walker, the sister species *U. stylata* and its specialist endoparasitoid *E. compressa* F. and its associated *E. robusta* were screened from individuals collected in Denmark and Germany in 2014 (Appendix S1). The endoparasitoids *E. serratulae* and *E. compressa* were analyzed as adults and treated as described above for *U. cardui*, whereas the three ectoparasitoid species were analyzed as both adults and larvae. The latter were identified to species with diagnostic isocitrate‐dehydrase (Idh) alleles (Johannesen & Seitz, 2003b).

**Table 1:** Locations and *Wolbachia* double infection in *Urophora cardui*. Double infections consist of *Urophora cardui*'s own strain and one associated with the endoparasitoid *Eurytoma serratulae*

Year	Locality	Coordinates	Country	Region	Es	*N*	SI_Ind_	DI_ind_	No Ampl.	DI
2001	Vium	56′37′N 08′52′E	DK	North	no	12	9	3	0	0.25
2001	Spøttrup	56′39′N 08′47′E	DK	North	no	3	2	0	1	0.00
2001	Brande	55′57′N 09′07′E	DK	North	no	3	3	0	0	0.00
2001	Kolding	55′32′N 09′27′E	DK	North	no	12	8	4	0	0.33
2001	Vildbjerg	56′11′N 08′45′E	DK	North	no	12	7	5	0	0.42
2001	Vind	56′14′N 08′32′E	DK	North	no	12	7	5	0	0.42
2001	Bodum	55′04′N 09′25′E	DK	Transition	no	15	10	4	1	0.27
2001	Christiansfeld	55′21′N 09′29′E	DK	Transition	no	3	1	2	0	0.67
2001	Frøslev	54′49′N 09′20′E	DK	Transition	no	11	8	3	0	0.27
2001	Oksekær	54′55′N 09′23′E	DK	Transition	no	11	11	0	0	0.00
2001	Ensted Kirke	55′01′N 09′24′E	DK	Transition	no	12	9	2	1	0.17
2001	Genner	55′13′N 09′28′E	DK	Transition	no	12	9	3	0	0.25
2001	Lottorf	54′28′N 09′34′E	D	South	no	12	10	2	0	0.17
2001	Neumünster	54′07′N 09′55′E	D	South	no	12	11	1	0	0.08
2001	Garlstorf	53′14′N 10′07′E	D	South	yes	14	11	3	0	0.21
2002	Struppen	50′56′N 14′00′E	D	Southeast	yes	6	5	1	0	0.17
2002	Eichelborn	50′57′N 11′12′E	D	Southeast	yes	5	5	0	0	0.00
2002	Oberberg	47′07′N 11′12′E	A	Southeast	no	5	5	0	0	0.00
2003	Sophienburg	49′56′N 11′36′E	D	Southeast	yes	1	1	0	0	0.00
2001	Obernschruz	49′23′N 12′42′E	D	Southeast	yes	7	7	0	0	0.00
2002	Schlegel	50′29′N 13′07′E	D	Southeast	yes	6	4	2	0	0.33
2002	Klence	49′26′N 12′49′E	CZ	Southeast	yes	7	5	0	2	0.00
2002	Rust	48′16′N 07′43′E	D	Southwest	yes	1	0	1	0	1.00
2002	Lay	50′22′N 07′34′E	D	Southwest	yes	4	4	0	0	0.00
2003	Dole	47′07′N 05′29′E	F	Southwest	yes	11	2	6	3	0.55
2003	Foussemange	47′38′N 06′58′E	F	Southwest	yes	15	10	2	3	0.13
2003	Montfaucon	47′14′N 06′05′E	F	Southwest	yes	5	5	0	0	0.00
2002	Nonnenweiher	49′37′N 07′07′E	F	Southwest	yes	2	0	2	0	1.00
2003	Lanans	47′18′N 06′37′E	F	Southwest	yes	7	5	2	0	0.29
2003	Bouclans	47′15′N 06′14′E	F	Southwest	yes	15	10	4	1	0.27
2003	Helsinki	60′10′N 23′56′E	SF	East	yes	7	7	0	0	0.00
2003	Kirov	58′33′N 49′39′E	RU	East	yes	14	13	0	1	0.00
2003	Tetujshi	54′59′N 48′49′E	RU	East	yes	13	10	3	0	0.23
2003	Yelabuga	54′46′N 52′06′E	RU	East	yes	5	4	1	0	0.20
2003	Yelets	52′39′N 38′29′E	RU	East	yes	9	8	1	0	0.11
2003	Kiev	50′19′N 30′24′E	UKR	East	yes	4	1	3	0	0.75
2003	Shropshire	52′23′N 02′20′W	GB	GB	yes	5	5	0	0	0.00
2003	Juniper Bottom	51′13′N 00′21′W	GB	GB	no	5	5	0	0	0.00
2003	Worcestershire	52′24′N 02′22′W	GB	GB	yes	5	5	0	0	0.00
2014	Vium	56′37′N 08′52′E	DK	North	yes	10	8	2	0	0.20
2013	Vildbjerg	56′11′N 08′45′E	DK	North	yes	19	7	12	0	0.63
2013	Kolding	55′32′N 09′27′E	DK	North	yes	9	6	3	0	0.33
2013/14	Vind	56′14′N 08′32′E	DK	North	yes	13	10	3	0	0.23
2013	Christiansfeld	55′21′N 09′29′E	DK	Transition	yes	13	6	7	0	0.54
2013	Ustrup Øst	55′21′N 09′38′E	DK	Transition	yes	12	7	3	2	0.25
2013	Marstrup	55′20′N 09′54′E	DK	Transition	yes	7	1	5	1	0.71
2014	Stollingvej	55′07′N 09′43′E	DK	Transition	yes	11	2	7	2	0.64
2013	Østre Løgum	55′11′N 09′36′E	DK	Transition	yes	23	0	23	0	1.00
2013	Frøslev	54′49′N 09′20′E	DK	Transition	yes	13	5	6	2	0.46
2013	Neumünster	54′07′N 09′55′E	D	South	yes	15	15	0	0	0.00
2014	Lottorf	54′28′N 09′34′E	D	South	yes	12	8	0	4	0.00
2013/14	Garlstorf	53′14′N 10′07′E	D	South	yes	13	10	3	0	0.23
2014	Genthin	52′40′N 12′11′E	D	Central Germany	yes	19	4	7	8	0.37
2014	Rhüden	51′57′N 10′08′E	D	Central Germany	yes	11	8	3	0	0.27
Total						520	339	149	32	

Es, *E. serratulae* present in sample year; N, number of samples tested for double infection per location; DI_ind_, individuals with double infection; SI_Ind_, individuals with single infection (*U. cardui* strain), the proportion of double infection; DI, is based on positive amplifications only.

GB, Great Britain (England); F, France; DK, Denmark; D, Germany; A, Austria; CZ, Czech Republic; SF, Finland; UKR, Ukraine; RU, Russia.

Differences in the frequency of DI were tested among temporal (2001 vs. 2013/14) and geographic (regions) samples, and between sexes of *U. cardui* (sexes only for 2013/14, data from 2001 to 2003 lost) with Fisher's exact tests using the online application vassarstats (http://vassarstats.net/). I assessed whether the presence of *E. serratulae* influenced DI in *U. cardui* at the local population level by regressing *E. serratulae* parasitization rates against *Wolbachia* DI proportions, and at individual level by analyzing whether presence/absence of the parasitoid in galls influenced DI of flies in these galls (Fisher's exact tests).

### 2.2 DNA and MLST procedures

DNA was extracted from individual specimens with High Pure PCR Template Preparation Kit (Roche Diagnostics) according to the manufacturer's protocol. DNA concentrations and 260/280 ratios were measured before PCR with NanoDrop^®^ (PEQLAB, Erlangen, Germany). Primers and thermocycler protocols for MLST genes (gatB, CoxA, hcpA, fstZ, fbpA) and wsp were as described in Baldo et al. (2006). I further sequenced the gene AAT. AAT has been proposed as an additional MLST gene (Paraskevopoulos, Bordenstein, Wernegreen, Werren, & Bourtzis, 2006), but it is also the primary gene defining the transition zone in *U. cardui* (Steinmetz et al., 2004). Hence, apart from delimiting *Wolbachia* strains, AAT sequence analysis also assessed whether the transition zone in *U. cardui* was influenced by *Wolbachia* allele diversity rather than by its own, nuclear diversity. AAT was amplified with the primers aspC49‐F (5′‐ATYGCTGTRACYGATAAGGYAA) and aspC1134‐R (5′‐AGARGTWGCATAAGARATTCTRA) (Paraskevopoulos et al., 2006) using the wsp PCR amplification protocol. Each PCR reaction was performed in 25 μl reaction volume consisting of 1 μl forward and 1 μl reverse primer, 12.5 μl OneTaq 2X Master Mix (NEB) and 9.5 μl H_2_O (sterile) and 1 μl DNA. PCR products were purified using NucleoSpin Extract II kits (Macherey & Nagel, Düren, Germany). Purified PCR products were Sanger sequenced at StarSeq^®^Gmbh (Mainz, Germany). Sequences were aligned with Sequence Navigator (ABI). Subsequently, all aligned sequences were checked manually.

Full MLST of *Wolbachia* strains associated with *U. cardui* (*N* = 4) and *E. serratulae* (*N* = 4) was obtained from individuals of each species that originated from at the same localities in Great Britain, Denmark, Germany, and Russia. In addition to the five MLST loci, I sequenced the wsp gene from 18 *E. serratulae* and 16 *U. cardui* and the gene AAT from six *E. serratulae* and seven *U. cardui* across the distribution range.

The phylogenetic positions of the *U. cardui* and *E. serratulae*‐associated *Wolbachia* strains were analyzed using MLST genes within the A supergroup. A total of 129 supergroup A strains with full MLST were downloaded from the *Wolbachia* MLST database (http://pubmlst.org/wolbachia/), aligned with the DI strains Ucar_A1 and Eser_A (see Section 3) using Muscle (Edgar, 2004) in the EMBI‐EBI tools framework (Li et al., 2015). Phylogenetic analysis was performed with the Kimura two‐parameter model and neighbor‐joining tree construction using Mega 6.0.6 (Tamar, Stecher, Peterson, Filipski, & Kamar, 2013).

### 2.3 Procedure for testing mixed infections

For identification of *Wolbachia* DI in *U. cardui*, specific wsp forward primers were designed from signature oligo‐nucleotide sequences located in HVR1 (position 19‐40) in *U. cardui* and in HVR 2 (173‐192) in *E. serratulae*. These variants correspond to HVR1 allele 53 and HVR2 allele 9 (MLST website, Jolley & Maiden, 2010). The specific forward primer sequences were 5′‐GCACATAAATCAGGCAAAGACA‐3′ (Uc1‐F) for *U. cardui* and 5′‐CGCCAGATACTATTGCAGAC‐3′ (Es2‐F) for *E. serratulae*. Amplifications of individual specimens were performed in 25 μl reaction volume consisting of 0.5 μl Uc1‐F, 0.5 μl Es2‐F, 0.5 μl wsp 691R, 12.5 μl OneTaq 2X Master Mix and 10.0 μl sterile H_2_O and 1 μl DNA. PCR products were amplified with the standard wsp amplification protocol (Baldo et al., 2006). All negative and ambiguous amplifications were tested twice.

I assessed whether *Wolbachia* DI in *U. cardui* involved the *E. serratulae* strain rather than another, albeit similar strain, using two strategies. First, I sequenced double‐infected *U. cardui*. *Eurytoma serratulae* has a hitherto new, specific gatB allele that differs at position 360 (G vs. C) (see Section 3). DI individuals must have clear G/C at this position. Second, I searched for *Wolbachia* MLST genes and mtDNA CO1 sequences from *U. cardui* (FJ713112) and *E. serratulae* (KU555516/17, this study) (*c*. 21% divergence) in an Illumina Miseq library generated from one DI male *U. cardui* (BioProject: PRJNA352663; SRA: SRR5023787). Presence of *U. cardui* CO1 in combination with the absence of *E. serratulae* CO1 confirms presence of the CO1 sequence, that is, of *U. cardui*, but shows the absence of *E. serratulae*‐mtDNA in this DI individual.

### 2.4 Wolbachia in other species associated with *Urophora cardui*

Screening for *Wolbachia* infection in other species than *U. cardui* and *E. serratulae* was performed with wsp and CoxA (Baldo et al., 2006) and always included a positive sample. To guarantee DNA quality in *Wolbachia* negative individuals, I amplified host COI using the primers C1‐N‐2776 and C1‐J‐1751 or C1‐J‐1718 (Hedin & Maddison, 2001; Simon et al., 1994) in 25 μl reaction volume consisting of 1 μl forward and 1 μl reverse primer, 12.5 μl OneTaq 2X Master Mix, and 9.5 μl H_2_O.

## 3 Results

### 3.1 Specificity of primers for detection of mixed infection

**Figure 3:**
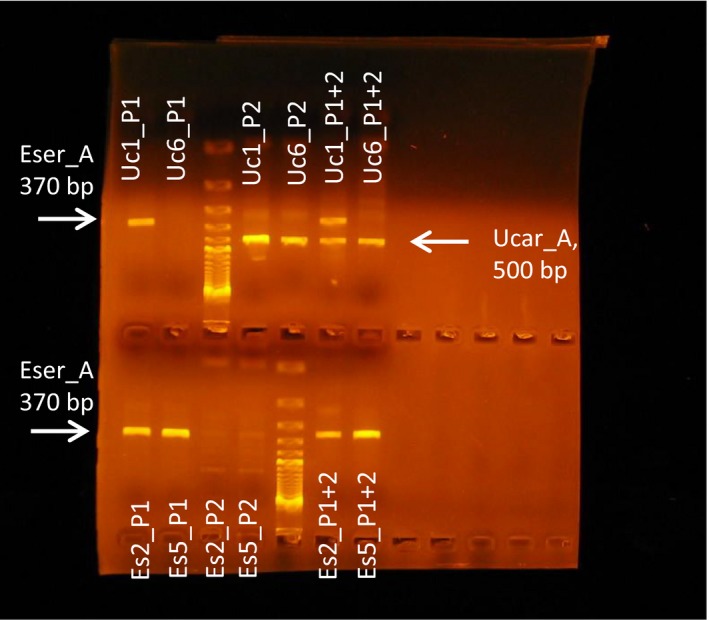
*Wsp* amplification patterns of *Wolbachia* strains with specific primers. *Eurytoma serratulae* (Eser_A) strain = 370 bp, *Urophora cardui* (Ucar_A1/A2) strain(s) = 500 bp. Two individuals of *E. serratulae* (bottom: Es2, Es5) and of *U. cardui* (top: Uc1, Uc6) were amplified with the F‐primers specific for *E. serratulae* (P1) and *U. cardui* (P2) strains, respectively, or with both F‐primers (P1+2). Uc1 is double‐infected and Uc6 is single infected. *E. serratulae* (Es) are always single infected.

Figure 3 shows amplification patterns of the specific primers for two individuals of each species with different primer combinations. The specific F‐primers Uc1‐F and Es2‐F amplified the *Wolbachia* strains associated with the fly (370 bp) and parasitoid (500 bp), respectively. In combination, the specific primers amplified both strains in individuals with DI (U1_P1+2) but never the alternative strain in single infected individuals (U6_P1+2, S2_P1+2, S5_P1+2). In many individuals of both species, shadow bands about 150–170 bp shorter than the target sequence were observed. The length difference between fly‐shadow (c. 320 bp) and the parasitoid‐target (370 bp) was about 50 bp and easily discernible both in length and intensity (see e.g., U1_P1+2).

### 3.2 MLST

MLST profiles are presented in Table 2. New alleles were found for CoxA (allele 236, *U. cardui* strain), hcpA (allele 282, *U. stylata* strain), and gatB (allele 254, *E. serratulae* strain). Two specific *U. cardui* strains (Ucar_A1, Ucar_A2) were observed. The strains differed at hcpA only, showing allele 42 (Western Europe) and 40 (Russia). The former hcpA allele belongs to supergroup A whereas the latter belongs to supergroup B. Post hoc sequence analysis of hcpA (*N* = 7) found allele 40 in all the Russian localities (*N* = 5) but not in Kiev (Ukraine) and Helsinki (Finland) (*N* = 2), which both had allele 42. The *E. serratulae* strain (Eser_A) was supergroup A, except for WSP‐HVR2, which was supergroup B (Table 2). The *U. stylata* strain (Usty_B) belonged to supergroup B.

**Table 2:** *Wolbachia* MLST profiles. hcpA in *Urophora cardui*‐E and HVR2 in *Eurytoma serratulae* represent putative recombination events of which only the former is related to the species in question

Host species	Strain	*N*	Gene, Profile					wsp	HVR1	HVR2	HVR3	HVR4
			gatB	coxA	hcpA	ftsZ	fbpA					
*U. cardui*	Ucar_A1	3	32	236	42	154	122	311	53	145	39	18
*U. cardui*	Ucar_A2	1	32	236	40^a^	154	122	311	53	145	39	18
*U. stylata*	Usty_B	2	9	9	282	8	10	63	19	17	24	33
*E. serratulae*	Eser_A	4	254	7	198	3	1		17	9^a^	22	18

*N*, number of individuals analyzed.

a Supergroup B allele.

Sequence analysis of gatB in DI individuals showed G/C double peaks at the Eser_A diagnostic site, confirming DI by this strain (Appendix S2). Further sequence analyses of wsp, fbpA, and CoxA from DI individuals confirmed DI with Eser_A (results not shown). The alleles associated with strains Ucar_A1, Ucar_A2, Eser_A, and Usty_B have GenBank accession numbers: AAT (aspC), KU647001‐ KU647004; fstZ, KU647005‐ KU647008; gatB, KU647009‐ KU647012; hcpA, KU647013‐16; wsp, KU647017 KU647020; coxA, KU647021‐ KU647024; fbpA, KU647025‐28.

BLASTN searches for Eser_A MLST genes in *U. cardui* reads identified strain‐specific partial sequences with 100% identity at all loci of both strains. For fbpA, the full 470‐bp Eser_A sequence was found, while the remaining genes were represented by 138–199 bp‐specific contigs (Appendix S3). BLASTN analysis of CO1 sequences retrieved CO1 from *U. cardui* only. The BLASTN analysis also retrieved *Wolbachia* and *U. cardui*‐like CO1 contigs that were not represented in NCBI and *Wolbachia* databases (1–3% divergence/contig) (search: 15.12.2015). All alternative sequences had open reading frames*. Post hoc* analysis of hypothetical alternative MLST sequences ruled out an erroneous contig assembly based on a combination (or recombination) of the two *Wolbachia* strains.

**Figure 4:**
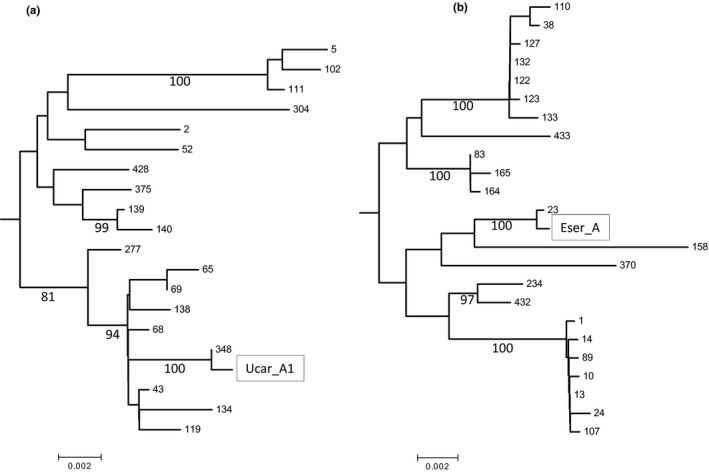
Phylogenetic positions of Ucar_A1 (A) and Eser_A (B) within subtrees of supergroup A. The positions of each subtree within the full phylogenetic tree (131 taxa) based on Neighbor‐Joining are presented in Appendix S4. Numbers at branch tips refer to MLST strains. Numbers below branches show bootstrap scores above 80

A phylogenetic tree based on 2071 positions in the final data set (gaps deleted) of 131 *Wolbachia* MLST strains had an optimal tree of branch length = 0.67672. Ucar_A1 was most related to ST 348 (identity 99.9%) while Eser_A was most related to ST23 (99.9%). The genetic identity between Ucar_A1 and Eser_A was 97.1%. Phylogenetic subtrees for each strain are presented in Figure 4, and their positions within the full phylogenetic tree are presented in Appendix S4.

### 3.3 Infection of *Urophora cardui*

*Wolbachia* was detected in 94% of all *U. cardui* across all populations and years (*N* = 520). DI was observed in all geographic regions, except GB (*N* = 15) (Table 1), thus rejecting the hypothesis that DI in Jutland was acquired after immigration of *E. serratulae*. Eser_A was never detected alone, always together with Ucar_A, suggesting that the former was not dominant invasive in *U. cardui*. DI in 2013–2014 was observed at equal proportions in males (45 DI, 38 SI, 8 no ampl.) and females (43 DI, 39 SI, 11 no ampl.) (NB. remaining four adults not sexed + 12 larvae). The DI proportion of positive individuals ranged between 0.03 in southeast Germany (2002–2003) and 0.71 in the transition region (2013–2014). DI was present in *U. cardui* before the immigration of *E. serratulae* on the Jutland peninsula in the second half of the 2000's. For example, the proportion in the northern and isolated population Vind, where *E. serratulae* was first observed in 2014, was 0.42 in 2001 (*N* = 12). In 2001, DI proportions did not differ among the regions north (0.31), within (0.25) and south (0.16), of the transition zone, all *p* > .20. DI did not differ significantly between 2001 and 2013/14 in regions north (0.31 and 0.37), *p* > .67, and south (0.16 and 0.08), *p* > .48. By contrast, DI increased significantly in the transition region between 2001 (0.25) and 2013/14 (0.71), *p* < .001.

The proportion of DI in *U. cardui* was related neither to the *E. serratulae* population parasitization rate (*r* ^2^ = .006, *p* > .50) (Appendix S5) nor to the probability of a gall being attacked by a parasitoid. The latter was evidenced in three ways: (1) the presence of DI in populations lacking *E. serratulae* in 2001, (2) no relationship in the intermediate parasitized and DI population Vildbjerg (parasitization 0.43, DI 0.42), and (3) no relationship in the low parasitized but completely DI population Østre Løgum (parasitization 0.09, DI 1.00).

### 3.4 Infection in associated species

*Wolbachia* was observed in all investigated *E. serratulae* (*N* = 45) and *U. stylata* (*N* = 20) but not in other parasitoid species associated with *U. cardui* (*E. robusta, N* = 35; *Tomyrus* sp., *N* = 12; *P. elevatus*,* N* = 12) or in those associated with the sister species *U. stylata* (*E. compressa*,* N* = 17; *E. robusta*,* N* = 16). Sequence analysis of the *wsp* gene for 18 individuals and MLST for four individuals in *E. serratulae* from throughout the distribution range showed no signs of DI in this species.

## 4 Discussion

Fitness effects of *Wolbachia* on insects are experimentally well documented and diverse (Werren, 1997), yet elucidation of horizontal transmission between specific species in natural populations is often hampered by the difficulty of obtaining temporal or geographic data that relates environment to infection status, where the strongest support comes from immigrant species acquiring new, resident strains (e.g., Schuler et al., 2013). In the present survey, I used these settings to study the history of DI in a dipteran host, *U. cardui*, and the evidence for transmission via the monophague endoparasitoid *E. serratulae*. Infection with the parasitoid strain Eser_A in the fly host in an area previously lacking the parasitoid was not related to immigration of said species, and immigration also did not lead to transmission of *Wolbachia* Ucar_A1 from *U. cardui* to the parasitoid there. DI in *U. cardui* was found in geographically and genetically separated populations with different intrataxon strains. As the phylogeographic differentiation in *U. cardui* is found both for mtDNA and nuclear genes (Johannesen et al., 2010), the present study provides evidence for sustained DI in *U. cardui* that is older than the current geographic distribution of the fly and which is not related to a selective sweep of infected mitochondrial haplotypes (Hurst & Jiggins, 2005). The genetic transition zone in *U. cardui*, which is defined by allelic variation of AAT, was not related to *Wolbachia* AAT.

### 4.1 History of DI in Urophora cardui

*Wolbachia* DI is found in numerous species and often includes both supergroup A and B strains (Werren, 1998). Theoretical studies have highlighted that the duration of DI will depend on CI and/or the geographic context of infection (Hurst & Jiggins, 2005; Werren, 1998). DI individuals may be incompatible with single‐infected individuals (Mercot, Llorente, Jacques, Atlan, & Monchamp‐Moreau, 1995); alternatively, double‐infected individuals backcrossed to uninfected individuals can cause segregation into mono‐infections (Poinsot, Montchamp‐Moreau, & Merçot, 2000). Such shifts in strain frequency can be extremely rapid (Kriesner et al., 2013; Schuler et al., 2013). In *U. cardui*, the own strain(s) were at or near fixation, while DI with Eser_A varied highly among populations. DI ratios were constant in two immigration regions while it increased in one region. The observation that both single and double‐infected individuals were equally represented in both sexes (1:1 sex ratio) at present indicates that neither incompatibility with single‐infected individuals nor sex ratio distortion effects are causing invasion of an alternative strain (Hurst, Jiggins, & Pomiankowski, 2002). Based on these considerations, there is presently no evidence from the distribution data that *Wolbachia* affects the genetic transition zone observed in *U. cardui* by reducing gene flow between the population groups, as observed in other species (Telschow, Engelstädter, Yamamura, Hammerstein, & Hurst, 2006; Werren, 1998). Rather, at present, the distribution data suggest that repeated colonization‐extinction dynamics typical for *U. cardui* (Eber & Brandl, 1996) induce regional variance in DI, but fitness‐related interactions cannot be ruled out. Hence, the initial observation of lack of DI south of the transition zone (see Section 1) was founded on variance in DI prevalence among regions and a low rate of DI in the region south in 2014, specifically.

The geographic distribution of DI and the absence of correlations with the degree of interaction with the parasitoid, including the absence of *E. serratulae*‐mtDNA (CO1) in the *U. cardui* Miseq data, contradict DI caused by encapsulation of parasitoid larvae within adult host flies. However, MiSeq data had sequences that differed slightly to the query sequences and which were found neither in NCBI nor in *Wolbachia* MLST databases. The diversity was found at two organismic levels, for mtDNA of the fly and *Wolbachia*. At present, it is not possible to determine whether these alternative sequences represent sequence errors, or additional and/or transient (lost) strain diversity as found in other tephritids (Arthofer et al., 2009; Morrow, Frommer, Royer, Shearman, & Riegler, 2015) and *Nasonia* wasps (Raychoudhury et al., 2009).

### 4.2 Ecological scenarios of acquisition

The presence of an identical strain in the monophague *E. serratulae* and its host *U. cardui* implies horizontal transmission between them. However, the infection pattern in the area of prior absence of the parasitoid did not support transmission from parasitoid to host there. An acquisition pattern between the two species, as described by the minimum number of required steps, has two scenarios with three steps each. In scenario one, each species acquired *Wolbachia* independently and was followed by transmission from *E. serratulae* to *U. cardui*. In scenario two, *U. cardui* experienced two acquisitions followed by transmission of one strain from *U. cardui* to *E. serratulae*. Evaluation of such scenarios requires comparative knowledge of external infection probabilities, which I evaluated using a community survey of species associated with *U. cardui*. Of the parasitoids associated with the sister species *U. cardui* and *U. stylata*, which had independent acquisitions, only *E. serratulae* was infected. Assuming that transmissions from other parasitoids, despite low sample sizes for these species, to their hosts are unlikely or can be excluded, transmission from the specialist *E. serratulae* to *U. cardui* might also be unlikely. This indirectly implies a pathway from fly to parasitoid. Mitochondrial DNA haplotypes of *E. serratulae* from Denmark and Russia (this study) were identical, thus indicating less mtDNA population structure in *E. serratulae* than *U. cardui*, where the former does not have a discrete east–west division. Combined with a male sex ratio of 0.35–0.40 in *E. serratulae* (Johannesen & Prill, unpublished data), the data are more in line with a recent *Wolbachia* sweep in *E. serratulae* than vice versa. Due to the singular presence of Eser_A in the parasitoid among geographic regions and its coexistence with fly‐specific strains in the fly, the acquisition of Eser_A by the parasitoid should be younger than that of the recombination event in the eastern fly *Wolbachia* strain Ucar_A2. From this follows that the parasitoid strain in *U. cardui* the fly might be the older and ancestral strain.

Notwithstanding the route of transmission of Eser_A between *E. serratulae* and *U. cardui* is the question from where and how it was introduced into the specialist *E. serratulae*‐*U. cardui* system. An intriguing notion is transmission via pupae and is based on the ecology of hosts harboring Eser_A's most related strains, which are found in the generalist parasitoids *Nasonia vitripennis* and *Muscidifurax raptor* (ST23). Both parasitoids are native to the eastern hemisphere and both probe and oviposite in puparia of fly hosts (Wylie, 1967). Although *U. cardui* pupae are concealed within a gall, gall deterioration in spring and/or gall opening by birds may expose puparia to various unspecific parasitoids (Vikberg, 2005). Transmission of an ancestor Eser_A strain to *U. cardui* puparia from a foreign parasitoid could happen to both parasitized (i.e., with *E. serratulae*) as well as unparasitized pupae. This scenario cannot decide the priority of first acquisition by *U. cardui* or *E. serratulae*, which theoretically may be simultaneous, but it does embed the transmission of *Wolbachia* in a niche context of oviposition strategy. It suggests that besides cophylogenetic mapping analysis (e.g., Ahmed et al., 2016), community level analysis of infection may help elucidate (or rule out) the biology of enigmatic transmissions across taxa.

The context of acquisition for the strains specific to *U. cardui* and *U. stylata* remains open. For the *U. cardui* strain Ucar_A1, the most related strain infests the weevil *Ceutorhynchus obstrictus* (2bp difference), which is associated with *Brassica* (cabbage). For *U. stylata*, the identical MLST stain (save for 1bp) has been found in three Lepidoptera, the European paper wasp, and the asparagus beetle parasitoid *Tetrastichus coeruleus* (MLST databank; Reumer, Van Alphen, & Kraaijeveld, 2010). This multihost pattern is typical for *Wolbachia*. For example, ST19 is found in three Lepidoptera, six species of ants, a weevil, and two species of braconid wasps. The range of similar strains among these diverse taxa is difficult to understand. Analyzing phylogenetic data, Ahmed et al. (2016) found evidence for specific shared food sources and shared natural enemies as possible routes of horizontal transmission in Lepidoptera, but there were no geographic correlations. Interestingly, both ST19 and ST23 have wasp hosts, which have become cosmopolitan recently. For ST19, it is the wasp *Cotesia* (*Apanteles*) *chilonis*, a widely used biocontrol agent (Fernandez‐Triana, Noyes, Polaszek, & Yu, 2015). Whether these wasps were the reservoirs of these shared strains worldwide or have become infected as a consequence of range expansion remains to be analyzed. The *U. cardui Wolbachia* system offers a possibility to address such quandaries through its recent establishment in North America and exposure to a new parasitoid community (De Clerck‐Floate & Cárcamo, 2011; Peschken & Derby, 1997).

Elaborating on these insights and in light of the pupae‐niche‐context transmission suggested for *U. cardui* and *E. serratulae*, I hypothesize that horizontal transmission across diverse taxa might be facilitated via generalist parasitoids infecting pupae during metamorphosis. A mechanistic liability that could promote horizontal acquisition during pupation is alteration of the actin cytoskeleton or filaments (Meulemans & de Loof, 1990). Complex cytoskeletal structures have more time to appear during late development and metamorphosis, being potentially less exposed to the disruptive effects of cell division in early differentiating cells (Jacinto & Baum, 2003). Newton, Savytskyy, and Sheehan (2015) found that *Wolbachia* rely on the actin cytoskeleton to achieve adequate titer in a *Drosophila* host during development and that regulation of actin is important to the maintenance of a *Wolbachia* infection during development. Melnikow et al. (2013) showed that WSP family proteins might be involved in optimization of the energy production pathway as well as in anchoring *Wolbachia* to the host's cytoskeleton. The hypothesis entails that pupae represent the most receptive stage for horizontal infections, which can be tested as the (trans)infection probability among developmental stages (egg, larvae, early/late pupae) of potential or actual hosts.

## Conflict of Interest

None declared.
