# Supplementary material for: Tracing the history and ecological context of Wolbachia double infection in a specialist host (Urophora cardui)—parasitoid (Eurytoma serratulae) system
Source: Ecol Evol. 2017 Jan 17;7(3):986–96. doi: 10.1002/ece3.2713 (PMC5288247; doi:10.1002/ece3.2713)
Supplement: Supplementary file 1 [file ECE3-7-986-s001.docx]

**Appendix S1.** **Johannesen, J.: Tracing the history and ecological context of *Wolbachia* double infection in a specialist host (*Urophora cardui*) – parasitoid (*Eurytoma serratulae*) system**

Locations and sample sizes for *Wolbachia* infections in parasitoids associated with *Urophora cardui* and *U. stylata*. Es = *Eurytoma serratulae*, Er-Uc *E. robusta* associated with *U. cardui*, Er-Us = *E. robusta* associated with *U. stylata*, Ec = *E. compressa*, Pe = *Pteromalus elevatus,* Tc =*Torymus* *chloromerus*.

|  |  |  |  |  | Parasitoid | | | | | |
| --- | --- | --- | --- | --- | --- | --- | --- | --- | --- | --- |
| Year | Locality | Coordinates | Country^1^ | Region | Es | Er-Uc | Er-Us | Ec | Pe | Tc |
| 2014 | Vium | 56’68’N 08’94’E | DK | North |  |  |  |  | 1 | 1 |
| 2014 | N. Balling Kirke | 56’62’N 08’88’E | DK | North |  |  |  |  |  | 3 |
| 2014 | Rydhave | 56’45’N 08’75’E | DK | North |  |  |  |  | 1 |  |
| 2014 | Vind | 56’14’N 08’32’E | DK | North |  |  |  |  | 2 | 1 |
| 2013 | Vildbjerg | 56’11’N 08’45’E | DK | North | 3 | 3 |  |  |  |  |
| 2013 | Agård | 55’35’N 09’25’E | DK | North | 3 | 3 |  |  |  |  |
| 2013 | Kolding | 55’32’N 09’27’E | DK | North | 3 | 3 |  |  |  |  |
| 2014 | Vonsild | 55’45’N 09’47’E | DK | North |  |  |  |  | 1 |  |
| 2014 | Christiansfeld | 55’21’N 09’29’E | DK | Cline | 3 | 3 | 1 | 3 |  |  |
| 2013 | Ustrup Öst | 55’21’N 09’38’E | DK | Cline | 3 | 3 |  |  |  |  |
| 2013 | Marstrup | 55’20’N 09’54’E | DK | Cline |  |  |  |  |  |  |
| 2014 | Stollingvej | 55’07’N 09’43’E | DK | Cline |  |  |  |  |  |  |
| 2013 | Östre Lögum | 55’11’N 09’36’E | DK | Cline |  |  |  |  |  |  |
| 2014 | Kliplev | 54’94’N 09’40’E | DK | Cline |  |  |  |  |  | 1 |
| 2013 | Oksekær | 54’55’N 09’23’E | DK | Cline |  |  |  |  | 1 |  |
| 2013 | Froeslev | 54’49’N 09’20’E | DK | Cline | 3 | 3 |  |  |  |  |
| 2013 | Genner | 55’13’N 09’28’E | DK | Cline |  |  |  |  |  | 1 |
| 2013 | Neumünster | 54’07’N 09’55’E | D | South | 3 | 3 |  |  | 1 |  |
| 2014 | Lottorf | 54’28’N 09’34’E | D | South |  |  |  |  |  | 2 |
| 2014 | Schleswig | 54’52’N 09’50’E | D | South |  |  |  |  | 1 | 1 |
| 2013 | Garlstorf | 53’14’N 10’07’E | D | South | 3 |  |  |  |  |  |
| 2007 | Bayreuth | 49’56’N 11’36’E | D | SouthEast |  |  | 6 | 6 |  |  |
| 2002 | Eichelborn | 50’57’N 11’12’E | D | SouthEast | 2 |  |  |  |  |  |
| 2014 | Genthin | 52’40’N 12’11’E | D | Central |  |  |  |  | 1 | 3 |
| 2014 | Rhüden | 51’57’N 10’08’E | D | Central |  |  |  |  |  | 1 |
| 2014 | Waldgrehweiler | 49’66’N 07’74’E | D | SouthWest |  |  | 1 | 1 |  |  |
| 2003 | Foussemange | 47’38’N 06’58’E | F | SouthWest | 2 | 4 |  |  |  |  |
| 2002 | Nonnenweiher | 49’37’N 07’07’E | F | SouthWest | 2 |  |  |  |  |  |
| 2007 | Callbach | 49’43’N 07’41’E | D | SouthWest |  |  | 6 | 6 |  |  |
| 2014 | Callbach | 49’43’N 07’41’E | D | SouthWest |  |  | 2 | 1 |  |  |
| 2003 | Helsinki | 60’10’N 23’56’E | SF | East | 3 |  |  |  |  |  |
| 2003 | Kirov | 58’33’N 49’39’E | RU | East | 2 | 3 |  |  |  |  |
| 2003 | Tetujshi | 54’59’N 48’49’E | RU | East | 3 | 4 |  |  |  |  |
| 2003 | Yelabuga | 54’46’N 52’06’E | RU | East | 2 |  |  |  |  |  |
| 2003 | Yelets | 52’39’N 38’29’E | RU | East | 2 |  |  |  |  |  |
| 2003 | Kiev | 50’19’N 30’24’E | UKR | East | 1 |  |  |  |  |  |
| 2003 | Shropshire | 52’23’N 02’20’W | GB | GB | 2 | 1 |  |  |  |  |
| 2003 | Juniper Bottom | 51’13’N 00’21’W | GB | GB |  | 1 |  |  |  |  |
| 2003 | Worcestershire | 52’24’N 02’22’W | GB | GB |  | 1 |  |  |  |  |
| Total |  |  |  |  | 45 | 35 | 16 | 17 | 12 | 12 |

^1^ Country: GB = Great Britain (England), F = France, DK = Denmark, D = Germany, SF = Finland, UKR = Ukraine, RU = Russia.
