# Supplementary material for: Tracing the history and ecological context of Wolbachia double infection in a specialist host (Urophora cardui)—parasitoid (Eurytoma serratulae) system
Source: Ecol Evol. 2017 Jan 17;7(3):986–96. doi: 10.1002/ece3.2713 (PMC5288247; doi:10.1002/ece3.2713)
Supplement: Supplementary file 2 [file ECE3-7-986-s002.pptx]

## Slide 1
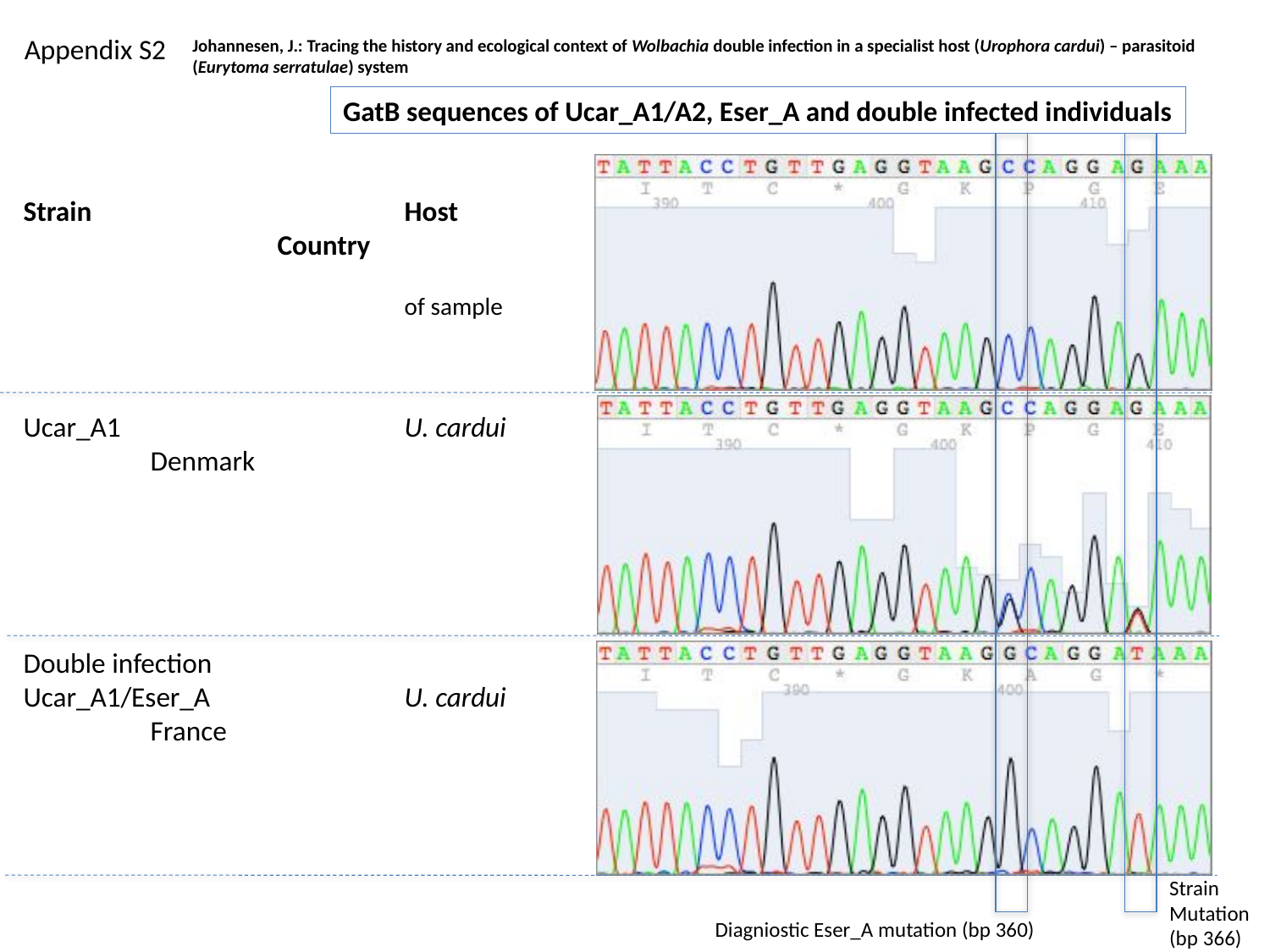

Appendix S2
Johannesen, J.: Tracing the history and ecological context of Wolbachia double infection in a specialist host (Urophora cardui) – parasitoid (Eurytoma serratulae) system
GatB sequences of Ucar_A1/A2, Eser_A and double infected individuals
Strain
Mutation
(bp 366)
Diagniostic Eser_A mutation (bp 360)
Strain			Host			Country
							of sample
Ucar_A1			U. cardui		Denmark
Double infection
Ucar_A1/Eser_A		U. cardui		France
Eser_A			E. serratulae	UK
