# Supplementary material for: Tracing the history and ecological context of Wolbachia double infection in a specialist host (Urophora cardui)—parasitoid (Eurytoma serratulae) system
Source: Ecol Evol. 2017 Jan 17;7(3):986–96. doi: 10.1002/ece3.2713 (PMC5288247; doi:10.1002/ece3.2713)
Supplement: Supplementary file 3 [file ECE3-7-986-s003.pdf]

Appendix S3.

Johannesen, J.: Tracing the history and ecological context of *Wolbachia* double infection in a specialist host (*Urophora cardui*) – parasitoid (*Eurytoma serratulae*) system

Blastn search for the Wolbachia Eser_A strain on a contig SOAPdeNovo (kmer=63) genome assembly of Urophora cardui generated from Illumina Miseq reads.

Query = Wolbachia strain from Eurytoma serratulae, Eser_A
Subject = contig retrieved from assembly.

The subject contigs are colour coded for sequences corresponding to strain

Subject Yellow = E. serratulae strain (U. cardui excluded)

Subject Green = U. cardui strain(E. serratulae excluded)

Subject Gray = other sequence

Subject Blue = not discernible between U. cardui and E. serratulae strains

```
BLASTN 2.2.30+


Reference: Zheng Zhang, Scott Schwartz, Lukas Wagner, and Webb
Miller (2000), "A greedy algorithm for aligning DNA sequences", J
Comput Biol 2000; 7(1-2):203-14.

Database: 120L_kmer63.contig
           11,496,826 sequences; 1,346,736,270 total letters
```

Query= fbpA_Wolbachia_Host_E.serratulae

```
Length=429
                                                                      Score     E
Sequences producing significant alignments:                          (Bits)  Value

  22763017 length 706 cvg_33.5_tip_0                                   793    0.0
  22022860 length 281 cvg_60.9_tip_0                                   399    2e-109
  21643576 length 235 cvg_1.0_tip_0                                    250    2e-64
  17623531 length 127 cvg_2.0_tip_0                                    180    2e-43
  18086659 length 127 cvg_2.0_tip_0                                    178    8e-43
  20753312 length 191 cvg_1.0_tip_1                                    165    6e-39
  19628033 length 148 cvg_63.0_tip_0                                   154    1e-35
  14504053 length 98 cvg_63.0_tip_0                                    143    3e-32
  13905850 length 92 cvg_63.0_tip_0                                    143    3e-32
  13865782 length 92 cvg_63.0_tip_0                                    135    5e-30
```

```
> 22763017 length 706 cvg_33.5_tip_0
Length=706

 Score =   793 bits (429),  Expect = 0.0
 Identities = 429/429 (100%), Gaps = 0/429 (0%)
 Strand=Plus/Minus

Query  1    GCTGGAATGCTCCCACTTATTTGAAACTTAATAGTTCCAACTCTTTACATTCAAAGGAT  60
            ||||||||||||||||||||||||||||||||||||||||||||||||||||||||||||
Sbjct  578  GCTGGAATGCTCCCACTTATTTGAAACTTAATAGTTCCAACTCTTTACATTCAAAGGAT  519

Query  61   CTAACCTCTGATCAGGCAATAACCTCTTCTGTGAAAGATGCGCTGCGTTTGGGATGCTTA  120
            ||||||||||||||||||||||||||||||||||||||||||||||||||||||||||||
Sbjct  518  CTAACCTCTGATCAGGCAATAACCTCTTCTGTGAAAGATGCGCTGCGTTTGGGATGCTTA  459

Query  121  GCTGTCGGATTTACTATATATCCTGGTTCTGCTAAGTGTTTCGATATGATGGAGGAAGCC  180
            ||||||||||||||||||||||||||||||||||||||||||||||||||||||||||||
Sbjct  458  GCTGTCGGATTTACTATATATCCTGGTTCTGCTAAGTGTTTCGATATGATGGAGGAAGCC  399

Query  181  CGTGGAATCATAGCTGAAGCCAAATCTTATGGACTTGCAGTAGTGCTATGGTCTTATCCA  240
            ||||||||||||||||||||||||||||||||||||||||||||||||||||||||||||
Sbjct  398  CGTGGAATCATAGCTGAAGCCAAATCTTATGGACTTGCAGTAGTGCTATGGTCTTATCCA  339

Query  241  CGCGGTGAAGGGATTTCCAAAGAAGGTGAAACAGCAGTTGATGTTATTGCCTATGCTGCG  300
            ||||||||||||||||||||||||||||||||||||||||||||||||||||||||||||
Sbjct  338  CGCGGTGAAGGGATTTCCAAAGAAGGTGAAACAGCAGTTGATGTTATTGCCTATGCTGCG  279

Query  301  CACATGGCAGCTTTGCTTGGCGCTAATATAATAAAAGTAAAACTTCCAACTAAATATTTG  360
            ||||||||||||||||||||||||||||||||||||||||||||||||||||||||||||
Sbjct  278  CACATGGCAGCTTTGCTTGGCGCTAATATAATAAAAGTAAAACTTCCAACTAAATATTTG  219

Query  361  GAAAGGGAGAAAATAGAAACAGAAAATATTGAATCATTATCTAAAAGAATTGAATATGTT  420
            ||||||||||||||||||||||||||||||||||||||||||||||||||||||||||||
Sbjct  218  GAAAGGGAGAAAATAGAAACAGAAAATATTGAATCATTATCTAAAAGAATTGAATATGTT  159

Query  421  AAAAGGTCT  429
            |||||||||
Sbjct  158  AAAAGGTCT  150


> 22022860 length 281 cvg_60.9_tip_0
Length=281

 Score =   399 bits (216),  Expect = 2e-109
 Identities = 250/267 (94%), Gaps = 0/267 (0%)
 Strand=Plus/Minus

Query  158  GTTTCGATATGATGGAGGAAGCCCGTGGAATCATAGCTGAAGCCAAATCTTATGGACTTG  217
            |||| ||||||||||| |||||||||| |||| |||||||||||||||||||||| ||||
Sbjct  281  GTTTTGATATGATGGAAGAAGCCCGTGAAATCGTAGCTGAAGCCAAATCTTATGGGCTTG  222

Query  218  CAGTAGTGCTATGGTCTTATCCACGCGGTGAAGGGATTTCCAAAGAAGGTGAAACAGCAG  277
            ||||||||||||||||||||||||| |||||||| |||||||||||||||||||||||||
Sbjct  221  CAGTAGTGCTATGGTCTTATCCACGTGGTGAAGGAATTTCCAAAGAAGGTGAAACAGCAG  162

Query  278  TTGATGTTATTGCCTATGCTGCGCACATGGCAGCTTTGCTTGGCGCTAATATAATAAAAG  337
            |||||||||||||||||||||||||||| |||||||| ||||||||||||||||| ||||
Sbjct  161  TTGATGTTATTGCCTATGCTGCGCACATAGCAGCTTTACTTGGCGCTAATATAATCAAAG  102

Query  338  TAAAACTTCCAACTAAATATTTGGAAAGGGAGAAAATAGAAACAGAAAATATTGAATCAT  397
            |||| |||||||||| |||||||||||  || || |||||||||||||||||||||||||
Sbjct  101  TAAAGCTTCCAACTAGATATTTGGAAAAAGAAAAGATAGAAACAGAAAATATTGAATCAT  42
```

```
Query  398  TATCTAAAAGAATTGAATATGTTAAAA  424
            |||||||||||||||||||| ||||||
Sbjct  41   TATCTAAAAGAATTGAATATATTAAAA  15


> 21643576 length 235 cvg_1.0_tip_0
Length=235

 Score =   250 bits (135),  Expect = 2e-64
 Identities = 171/189 (90%), Gaps = 0/189 (0%)
 Strand=Plus/Minus

Query  1    GCTGGAATGCTCCCACTTATTTTGAAACTTAATAGTTCCAACTCTTTACATTCAAAGGAT  60
            ||||||||||||||||||| |||||||| ||||||||||||||| |||||||||||  ||
Sbjct  191  GCTGGAATGCTCCCACTTACTTTGAAACATAATAGTTCCAACTCCTTACATTCAAAAAAT  132

Query  61   CTAACCTCTGATCAGGCAATAACCTCTTCTGTGAAAGATGCGCTGCGTTTGGGATGCTTA  120
            ||  | |||||||| ||||||||||||||||||||||| || ||||||||||| |||||
Sbjct  131  CTGGCTTCTGATCAAGCAATAACCTCTTCTGTGAAAGAAGCACTGCGTTTGGGCTGCTTG  72

Query  121  GCTGTCGGATTTACTATATATCCTGGTTCTGCTAAGTGTTTCGATATGATGGAGGAAGCC  180
            ||||| | ||||||||||||||||||||||||||||||||| ||||||||||| ||||||
Sbjct  71   GCTGTTGTATTTACTATATATCCTGGTTCTGCTAAGTGTTTTGATATGATGGAAGAAGCC  12

Query  181  CGTGGAATC  189
            |||| ||||
Sbjct  11   CGTGAAATC  3


> 17623531 length 127 cvg_2.0_tip_0
Length=127

 Score =   180 bits (97),  Expect = 2e-43
 Identities = 117/127 (92%), Gaps = 0/127 (0%)
 Strand=Plus/Plus
Query  94   AAAGATGCGCTGCGTTTGGGATGCTTAGCTGTCGGATTTACTATATATCCTGGTTCTGCT  153
            |||||||| ||||||||||| ||||| ||||| |||||||||||||||||||||||||||
Sbjct  1    AAAGATGCACTGCGTTTGGGCTGCTTGGCTGTTGGATTTACTATATATCCTGGTTCTGCT  60

Query  154  AAGTGTTTCGATATGATGGAGGAAGCCCGTGGAATCATAGCTGAAGCCAAATCTTATGGA  213
            ||| |||| ||||||||||| |||||||||| |||| ||||||||||||||||||||||
Sbjct  61   AAGCGTTTTGATATGATGGAAGAAGCCCGTGAAATCGTAGCTGAAGCCAAATCTTATGGG  120

Query  214  CTTGCAG  220
            |||||||
Sbjct  121  CTTGCAG  127


> 18086659 length 127 cvg_2.0_tip_0
Length=127

 Score =   178 bits (96),  Expect = 8e-43
 Identities = 116/126 (92%), Gaps = 0/126 (0%)
 Strand=Plus/Plus

Query  59   ATCTAACCTCTGATCAGGCAATAACCTCTTCTGTGAAAGATGCGCTGCGTTTGGGATGCT  118
            |||| || |||||||| |||||||||||||||||||||||||| ||||||||||| ||||
Sbjct  1    ATCTGACTTCTGATCAAGCAATAACCTCTTCTGTGAAAGATGCACTGCGTTTGGGCTGCT  60

Query  119  TAGCTGTCGGATTTACTATATATCCTGGTTCTGCTAAGTGTTTCGATATGATGGAGGAAG  178
            | | ||| ||||||||||||||||||||||||||||||||||| ||||||||||| ||||
Sbjct  61   TGGTTGTTGGATTTACTATATATCCTGGTTCTGCTAAGTGTTTTGATATGATGGAAGAAG  120
```

```
Query  179  CCCGTG  184
            ||||||
Sbjct  121  CCCGTG  126


> 20753312 length 191 cvg_1.0_tip_1
Length=191

 Score =   165 bits (89),  Expect = 6e-39
 Identities = 105/113 (93%), Gaps = 0/113 (0%)
 Strand=Plus/Minus

Query  1    GCTGGAATGCTCCCACTTATTTTGAAACTTAATAGTTCCAACTCTTTACATTCAAAGGAT  60
            |||||||||||||||||||||||||||||||||||||||||||| |||| ||||||  ||
Sbjct  113  GCTGGAATGCTCCCACTTATTTTGAAACTTAATAGTTCCAACTCCTTACCTTCAAAAAAT  54

Query  61   CTAACCTCTGATCAGGCAATAACCTCTTCTGTGAAAGATGCGCTGCGTTTGGG  113
            || || |||||||| |||||||||||||||||||||||||| |||||||||||
Sbjct  53   CTGACTTCTGATCAAGCAATAACCTCTTCTGTGAAAGATGCACTGCGTTTGGG  1


> 19628033 length 148 cvg_63.0_tip_0
Length=148

 Score =   154 bits (83),  Expect = 1e-35
 Identities = 95/101 (94%), Gaps = 0/101 (0%)
 Strand=Plus/Plus

Query  1    GCTGGAATGCTCCCACTTATTTTGAAACTTAATAGTTCCAACTCTTTACATTCAAAGGAT  60
            |||||||||||||||||||||||||||||||||||||||||||| |||||||||||  ||
Sbjct  45   GCTGGAATGCTCCCACTTATTTTGAAACTTAATAGTTCCAACTCCTTACATTCAAAAAAT  104

Query  61   CTAACCTCTGATCAGGCAATAACCTCTTCTGTGAAAGATGC  101
            || || |||||||| ||||||||||||||||||||||||||
Sbjct  105  CTGACTTCTGATCAAGCAATAACCTCTTCTGTGAAAGATGC  145


> 14504053 length 98 cvg_63.0_tip_0
Length=98

 Score =   143 bits (77),  Expect = 3e-32
 Identities = 91/98 (93%), Gaps = 0/98 (0%)
 Strand=Plus/Plus

Query  59   ATCTAACCTCTGATCAGGCAATAACCTCTTCTGTGAAAGATGCGCTGCGTTTGGGATGCT  118
            |||| || |||||||| |||||||||||||||||||||||||| ||||||||||| ||||
Sbjct  1    ATCTGACTTCTGATCAAGCAATAACCTCTTCTGTGAAAGATGCACTGCGTTTGGGCTGCT  60

Query  119  TAGCTGTCGGATTTACTATATATCCTGGTTCTGCTAAG  156
            | ||||| ||||||||||||||||||||||||||||||
Sbjct  61   TGGCTGTTGGATTTACTATATATCCTGGTTCTGCTAAG  98


> 13905850 length 92 cvg_63.0_tip_0
Length=92

 Score =   143 bits (77),  Expect = 3e-32
 Identities = 87/92 (95%), Gaps = 0/92 (0%)
 Strand=Plus/Plus

Query  129  ATTTACTATATATCCTGGTTCTGCTAAGTGTTTCGATATGATGGAGGAAGCCCGTGGAAT  188
            ||||||||||||||||||||||||||||||||| ||||||||||| |||||||||| |||
```

```
Sbjct  1    ATTTACTATATATCCTGGTTCTGCTAAGTGTTTTGATATGATGGAAGAAGCCCGTGAAAT  60

Query  189  CATAGCTGAAGCCAAATCTTATGGACTTGCAG  220
            | |||||||||||||||||||||| |||||||
Sbjct  61   CGTAGCTGAAGCCAAATCTTATGGGCTTGCAG  92


> 13865782 length 92 cvg_63.0_tip_0
Length=92

 Score =   135 bits (73),  Expect = 5e-30
 Identities = 85/91 (93%), Gaps = 0/91 (0%)
 Strand=Plus/Plus

Query  94   AAAGATGCGCTGCGTTTGGGATGCTTAGCTGTCGGATTTACTATATATCCTGGTTCTGCT  153
            |||||||| ||||||||||| ||||| ||||| |||||||||||||||||||||||||||
Sbjct  1    AAAGATGCACTGCGTTTGGGCTGCTTGGCTGTTGGATTTACTATATATCCTGGTTCTGCT  60

Query  154  AAGTGTTTCGATATGATGGAGGAAGCCCGTG  184
            |||||||| ||||||||||| ||||||||||
Sbjct  61   AAGTGTTTTGATATGATGGAAGAAGCCCGTG  91



Lambda      K        H
    1.33    0.621     1.12

Gapped
Lambda      K        H
    1.28    0.460    0.850

Effective search space used: 410954881942
```

Query= hcpA_Wolbachia_Host_E.serratulae

```
Length=444
                                                                      Score     E
Sequences producing significant alignments:                          (Bits)  Value

  22057630 length 287 cvg_1.0_tip_1                                   473   1e-131
  21830227 length 253 cvg_1.0_tip_1                                   446   2e-123
  20976622 length 199 cvg_36.7_tip_0                                  368   5e-100
  21427278 length 220 cvg_1.0_tip_1                                   340   1e-91
  20709012 length 190 cvg_1.0_tip_0                                   327   8e-88
  22043540 length 285 cvg_1.0_tip_1                                   263   2e-68
  21081276 length 203 cvg_1.0_tip_1                                   255   4e-66
  16987487 length 124 cvg_37.0_tip_0                                  230   2e-58
  19075283 length 132 cvg_60.3_tip_0                                  228   8e-58
  19569559 length 146 cvg_63.0_tip_0                                  226   3e-57
  15521461 length 111 cvg_33.4_tip_0                                  206   4e-51
  15424818 length 110 cvg_63.0_tip_0                                  204   1e-50
  14894712 length 103 cvg_60.8_tip_0                                  180   2e-43
  14221445 length 95 cvg_61.5_tip_0                                   176   3e-42
  16301770 length 119 cvg_63.0_tip_0                                  172   4e-41
  14962368 length 103 cvg_63.0_tip_0                                  163   2e-38
  13234477 length 87 cvg_61.3_tip_0                                   161   9e-38
  11246909 length 76 cvg_63.0_tip_0                                   134   2e-29
  10545799 length 73 cvg_42.0_tip_0                                   122   4e-26
  21173064 length 207 cvg_58.8_tip_0                                  117   2e-24
```

```
  21996609 length 277 cvg_1.0_tip_1                                   115   7e-24


> 22057630 length 287 cvg_1.0_tip_1
Length=287

 Score =   473 bits (256),  Expect = 1e-131
 Identities = 276/286 (97%), Gaps = 0/286 (0%)
 Strand=Plus/Plus

Query  5    CCGAACTCAACCCGCGCCTTCGCTCTGCTATATTTGCTGCACGCAAGGAAAATCTACCAA  64
            ||||||||||||||||||||||||||||||||||| ||||||||||||||||||||||||
Sbjct  1    CCGAACTCAACCCGCGCCTTCGCTCTGCTATATTTCCTGCACGCAAGGAAAATCTACCAA  60

Query  65   AAGATAAAATAGAAACAGCAATAAAAAATGCAACTGGTAACGTTGCTGGAGAAAATTACG  124
            ||||||||||||||||||||||||||||||||||||| |||||||||||||||||||| |
Sbjct  61   AAGATAAAATAGAAACAGCAATAAAAAATGCAACTGGCAACGTTGCTGGAGAAAATTATG  120

Query  125  AGGAAATACAATATGAAGGTCATGGGCCTTCTGGCACTGCACTCATTGTCCATGCCTTGA  184
            ||||||| ||||||||||||||||||||||||| ||||||||| ||||||||||||||||
Sbjct  121  AGGAAATCCAATATGAAGGTCATGGGCCTTCTGTCACTGCACTTATTGTCCATGCCTTGA  180

Query  185  CTAATAACCGCAACCGTACTGCTTCTGAGGTACGTTATATATTTTCTCGCAAGGGTGGAA  244
            |||||||||||||||||||||| |||| ||||||||||||||| ||||||||||||||||
Sbjct  181  CTAATAACCGCAACCGTACTGCGTCTGGGGTACGTTATATATTGTCTCGCAAGGGTGGAA  240

Query  245  ACTTGGGAGAAACAGGAAGTGTTAGTTACCTTTTTGATCATGTAGG  290
            |||||||||||||||||||||||||||||||||| |||||||||||
Sbjct  241  ACTTGGGAGAAACAGGAAGTGTTAGTTACCTTTTCGATCATGTAGG  286


> 21830227 length 253 cvg_1.0_tip_1
Length=253

 Score =   446 bits (241),  Expect = 2e-123
 Identities = 249/253 (98%), Gaps = 0/253 (0%)
 Strand=Plus/Plus

Query  36   ATTTGCTGCACGCAAGGAAAATCTACCAAAAGATAAAATAGAAACAGCAATAAAAAATGC  95
            ||||||||||||||||||||||||||||||||||||||||||||||||||||||||||||
Sbjct  1    ATTTGCTGCACGCAAGGAAAATCTACCAAAAGATAAAATAGAAACAGCAATAAAAAATGC  60

Query  96   AACTGGTAACGTTGCTGGAGAAAATTACGAGGAAATACAATATGAAGGTCATGGGCCTTC  155
            ||| ||||||||||||||||||||||||||||||||||||||||||||||||||||| ||
Sbjct  61   AACGGGTAACGTTGCTGGAGAAAATTACGAGGAAATACAATATGAAGGTCATGGGCCCTC  120

Query  156  TGGCACTGCACTCATTGTCCATGCCTTGACTAATAACCGCAACCGTACTGCTTCTGAGGT  215
            |||||||||||||||||||||||||||||||||||||||||| |||||||||||||||||
Sbjct  121  TGGCACTGCACTCATTGTCCATGCCTTGACTAATAACCGCAATCGTACTGCTTCTGAGGT  180

Query  216  ACGTTATATATTTTCTCGCAAGGGTGGAAACTTGGGAGAAACAGGAAGTGTTAGTTACCT  275
            |||||||||||||||||| |||||||||||||||||||||||||||||||||||||||||
Sbjct  181  ACGTTATATATTTTCTCGTAAGGGTGGAAACTTGGGAGAAACAGGAAGTGTTAGTTACCT  240

Query  276  TTTTGATCATGTA  288
            |||||||||||||
Sbjct  241  TTTTGATCATGTA  253


> 20976622 length 199 cvg_36.7_tip_0
Length=199

 Score =   368 bits (199),  Expect = 5e-100
```

```
 Identities = 199/199 (100%), Gaps = 0/199 (0%)
 Strand=Plus/Minus

Query  216   ACGTTATATATTTTCTCGCAAGGGTGGAAACTTGGGAGAAACAGGAAGTGTTAGTTACCT  275
             ||||||||||||||||||||||||||||||||||||||||||||||||||||||||||||
Sbjct  199   ACGTTATATATTTTCTCGCAAGGGTGGAAACTTGGGAGAAACAGGAAGTGTTAGTTACCT  140

Query  276   TTTTGATCATGTAGGTTTAATTGTCTATAAAGCAGAGGGTGTGAATTTTGATGATTTATT  335
             ||||||||||||||||||||||||||||||||||||||||||||||||||||||||||||
Sbjct  139   TTTTGATCATGTAGGTTTAATTGTCTATAAAGCAGAGGGTGTGAATTTTGATGATTTATT  80

Query  336   CAGTCATGGAATCGAATTAGAAGTATTGAATGTTGAGGAAAATGACAAAGAAGGATTACA  395
             ||||||||||||||||||||||||||||||||||||||||||||||||||||||||||||
Sbjct  79    CAGTCATGGAATCGAATTAGAAGTATTGAATGTTGAGGAAAATGACAAAGAAGGATTACA  20

Query  396   CGTTATAACTTGTGAAATA  414
             |||||||||||||||||||
Sbjct  19    CGTTATAACTTGTGAAATA  1


> 21427278 length 220 cvg_1.0_tip_1
Length=220

 Score =   340 bits (184),  Expect = 1e-91
 Identities = 208/220 (95%), Gaps = 0/220 (0%)
 Strand=Plus/Plus

Query  155   CTGGCACTGCACTCATTGTCCATGCCTTGACTAATAACCGCAACCGTACTGCTTCTGAGG  214
             ||||||||||||| ||||||||||||||||||||||||||||||||||||||| ||||||
Sbjct  1     CTGGCACTGCACTTATTGTCCATGCCTTGACTAATAACCGCAACCGTACTGCTCCTGAGG  60

Query  215   TACGTTATATATTTTCTCGCAAGGGTGGAAACTTGGGAGAAACAGGAAGTGTTAGTTACC  274
             |||||||||||||||||||||||||||||||||||| |||||||||||||||||||||||
Sbjct  61    TACGTTATATATTTTCTCGCAAGGGTGGAAACTTGGGCGAAACAGGAAGTGTTAGTTACC  120

Query  275   TTTTTGATCATGTAGGTTTAATTGTCTATAAAGCAGAGGGTGTGAATTTTGATGATTTAT  334
             |||| ||||||||||| ||||| ||||||||||||| |||||||||||||||||||||||
Sbjct  121   TTTTCGATCATGTAGGCTTAATCGTCTATAAAGCAGTGGGTGTGAATTTTGATGATTTAT  180

Query  335   TCAGTCATGGAATCGAATTAGAAGTATTGAATGTTGAGGA  374
             | | | |||| ||||| |||||||||||||||||||||||
Sbjct  181   TTAATTATGGGATCGAGTTAGAAGTATTGAATGTTGAGGA  220


> 20709012 length 190 cvg_1.0_tip_0
Length=190

 Score =   327 bits (177),  Expect = 8e-88
 Identities = 181/183 (99%), Gaps = 0/183 (0%)
 Strand=Plus/Minus

Query  1     CTACCCGAACTCAACCCGCGCCTTCGCTCTGCTATATTTGCTGCACGCAAGGAAAATCTA  60
             |||||||||||||||||||||||||||||||||||||||||||||||||||||||| |||
Sbjct  183   CTACCCGAACTCAACCCGCGCCTTCGCTCTGCTATATTTGCTGCACGCAAGGAAAAACTA  124

Query  61    CCAAAAGATAAAATAGAAACAGCAATAAAAAATGCAACTGGTAACGTTGCTGGAGAAAAT  120
             ||||||||||||||||||||||||||||||||||||||||||||||||||||||||||| 
Sbjct  123   CCAAAAGATAAAATAGAAACAGCAATAAAAAATGCAACTGGTAACGTTGCTGGAGAAAAA  64

Query  121   TACGAGGAAATACAATATGAAGGTCATGGGCCTTCTGGCACTGCACTCATTGTCCATGCC  180
             ||||||||||||||||||||||||||||||||||||||||||||||||||||||||||||
Sbjct  63    TACGAGGAAATACAATATGAAGGTCATGGGCCTTCTGGCACTGCACTCATTGTCCATGCC  4
```

```
Query  181  TTG  183
            |||
Sbjct  3    TTG  1


> 22043540 length 285 cvg_1.0_tip_1
Length=285

 Score =   263 bits (142),  Expect = 2e-68
 Identities = 148/151 (98%), Gaps = 0/151 (0%)
 Strand=Plus/Plus

Query  294  AATTGTCTATAAAGCAGAGGGTGTGAATTTTGATGATTTATTCAGTCATGGAATCGAATT  353
            ||||||||||||||||||||||||||||||||||||||||||||||||||||||||||||
Sbjct  1    AATTGTCTATAAAGCAGAGGGTGTGAATTTTGATGATTTATTCAGTCATGGAATCGAATT  60

Query  354  AGAAGTATTGAATGTTGAGGAAAATGACAAAGAAGGATTACACGTTATAACTTGTGAAAT  413
            || ||||||||||||||||||||||||||||||||| |||||||||||| ||||||||||
Sbjct  61   AGCAGTATTGAATGTTGAGGAAAATGACAAAGAAGGTTTACACGTTATACCTTGTGAAAT  120

Query  414  AAAAGATTTTGGTAAAGTACGCGATGCCTTT  444
            |||||||||||||||||||||||||||||||
Sbjct  121  AAAAGATTTTGGTAAAGTACGCGATGCCTTT  151


> 21081276 length 203 cvg_1.0_tip_1
Length=203

 Score =   255 bits (138),  Expect = 4e-66
 Identities = 152/159 (96%), Gaps = 0/159 (0%)
 Strand=Plus/Minus

Query  4    CCCGAACTCAACCCGCGCCTTCGCTCTGCTATATTTGCTGCACGCAAGGAAAATCTACCA  63
            ||| ||||||||||||||||||||||||||||||||||| |||||||||||||||||||||
Sbjct  159  CCCAAACTCAACCCGCGCCTTCGCTCTGCTATATTTGCAGCACGCAAGGAAAATCTACCA  100

Query  64   AAAGATAAAATAGAAACAGCAATAAAAAATGCAACTGGTAACGTTGCTGGAGAAAATTAC  123
            | ||| ||||||||||||||||||||||||||||| ||||||||||||||||||||||| 
Sbjct  99   AGAGAAAAAATAGAAACAGCAATAAAAAATGCAACGGGTAACGTTGCTGGAGAAAATTAT  40

Query  124  GAGGAAATACAATATGAAGGTCATGGGCCTTCTGGCACT  162
            |||||||| ||||||||||||||||||||||||||||||
Sbjct  39   GAGGAAATCCAATATGAAGGTCATGGGCCTTCTGGCACT  1


> 16987487 length 124 cvg_37.0_tip_0
Length=124

 Score =   230 bits (124),  Expect = 2e-58
 Identities = 124/124 (100%), Gaps = 0/124 (0%)
 Strand=Plus/Plus

Query  60   ACCAAAAGATAAAATAGAAACAGCAATAAAAAATGCAACTGGTAACGTTGCTGGAGAAAA  119
            ||||||||||||||||||||||||||||||||||||||||||||||||||||||||||||
Sbjct  1    ACCAAAAGATAAAATAGAAACAGCAATAAAAAATGCAACTGGTAACGTTGCTGGAGAAAA  60

Query  120  TTACGAGGAAATACAATATGAAGGTCATGGGCCTTCTGGCACTGCACTCATTGTCCATGC  179
            ||||||||||||||||||||||||||||||||||||||||||||||||||||||||||||
Sbjct  61   TTACGAGGAAATACAATATGAAGGTCATGGGCCTTCTGGCACTGCACTCATTGTCCATGC  120

Query  180  CTTG  183
            ||||
Sbjct  121  CTTG  124
```

```
> 19075283 length 132 cvg_60.3_tip_0
Length=132

 Score =   228 bits (123),  Expect = 8e-58
 Identities = 129/132 (98%), Gaps = 0/132 (0%)
 Strand=Plus/Minus

Query  100   GGTAACGTTGCTGGAGAAAATTACGAGGAAATACAATATGAAGGTCATGGGCCTTCTGGC  159
             ||||||||||||||||||||||| |||||||| |||||||||||||||||||||||||||
Sbjct  132   GGTAACGTTGCTGGAGAAAATTATGAGGAAATCCAATATGAAGGTCATGGGCCTTCTGGC  73

Query  160   ACTGCACTCATTGTCCATGCCTTGACTAATAACCGCAACCGTACTGCTTCTGAGGTACGT  219
             |||||||| |||||||||||||||||||||||||||||||||||||||||||||||||||
Sbjct  72    ACTGCACTTATTGTCCATGCCTTGACTAATAACCGCAACCGTACTGCTTCTGAGGTACGT  13

Query  220   TATATATTTTCT  231
             ||||||||||||
Sbjct  12    TATATATTTTCT  1


> 19569559 length 146 cvg_63.0_tip_0
Length=146

 Score =   226 bits (122),  Expect = 3e-57
 Identities = 138/146 (95%), Gaps = 0/146 (0%)
 Strand=Plus/Minus

Query  229   TCTCGCAAGGGTGGAAACTTGGGAGAAACAGGAAGTGTTAGTTACCTTTTTGATCATGTA  288
             |||||||||||||||||||||||||||||||||||||||||||||||||| |||||||||
Sbjct  146   TCTCGCAAGGGTGGAAACTTGGGAGAAACAGGAAGTGTTAGTTACCTTTTCGATCATGTA  87

Query  289   GGTTTAATTGTCTATAAAGCAGAGGGTGTGAATTTTGATGATTTATTCAGTCATGGAATC  348
             || ||||| |||||||||||||||||||||||||||||||||||||| | | |||| |||
Sbjct  86    GGCTTAATCGTCTATAAAGCAGAGGGTGTGAATTTTGATGATTTATTTAATTATGGGATC  27

Query  349   GAATTAGAAGTATTGAATGTTGAGGA  374
             || |||||||||||||||||||||||
Sbjct  26    GAGTTAGAAGTATTGAATGTTGAGGA  1


> 15521461 length 111 cvg_33.4_tip_0
Length=111

 Score =   206 bits (111),  Expect = 4e-51
 Identities = 111/111 (100%), Gaps = 0/111 (0%)
 Strand=Plus/Minus

Query  121   TACGAGGAAATACAATATGAAGGTCATGGGCCTTCTGGCACTGCACTCATTGTCCATGCC  180
             ||||||||||||||||||||||||||||||||||||||||||||||||||||||||||||
Sbjct  111   TACGAGGAAATACAATATGAAGGTCATGGGCCTTCTGGCACTGCACTCATTGTCCATGCC  52

Query  181   TTGACTAATAACCGCAACCGTACTGCTTCTGAGGTACGTTATATATTTTCT  231
             |||||||||||||||||||||||||||||||||||||||||||||||||||
Sbjct  51    TTGACTAATAACCGCAACCGTACTGCTTCTGAGGTACGTTATATATTTTCT  1


> 15424818 length 110 cvg_63.0_tip_0
Length=110

 Score =   204 bits (110),  Expect = 1e-50
 Identities = 110/110 (100%), Gaps = 0/110 (0%)
```

```
 Strand=Plus/Minus

Query  169  ATTGTCCATGCCTTGACTAATAACCGCAACCGTACTGCTTCTGAGGTACGTTATATATTT  228
            ||||||||||||||||||||||||||||||||||||||||||||||||||||||||||||
Sbjct  110  ATTGTCCATGCCTTGACTAATAACCGCAACCGTACTGCTTCTGAGGTACGTTATATATTT  51

Query  229  TCTCGCAAGGGTGGAAACTTGGGAGAAACAGGAAGTGTTAGTTACCTTTT  278
            ||||||||||||||||||||||||||||||||||||||||||||||||||
Sbjct  50   TCTCGCAAGGGTGGAAACTTGGGAGAAACAGGAAGTGTTAGTTACCTTTT  1


> 14894712 length 103 cvg_60.8_tip_0
Length=103

 Score =   180 bits (97),  Expect = 2e-43
 Identities = 101/103 (98%), Gaps = 0/103 (0%)
 Strand=Plus/Plus

Query  60   ACCAAAAGATAAAATAGAAACAGCAATAAAAAATGCAACTGGTAACGTTGCTGGAGAAAA  119
            ||||||||||||||||||||||||||||||||||||||||||||||||||||||||||||
Sbjct  1    ACCAAAAGATAAAATAGAAACAGCAATAAAAAATGCAACTGGTAACGTTGCTGGAGAAAA  60

Query  120  TTACGAGGAAATACAATATGAAGGTCATGGGCCTTCTGGCACT  162
            ||| |||||||| ||||||||||||||||||||||||||||||
Sbjct  61   TTATGAGGAAATCCAATATGAAGGTCATGGGCCTTCTGGCACT  103


> 14221445 length 95 cvg_61.5_tip_0
Length=95

 Score =   176 bits (95),  Expect = 3e-42
 Identities = 95/95 (100%), Gaps = 0/95 (0%)
 Strand=Plus/Minus

Query  4   CCCGAACTCAACCCGCGCCTTCGCTCTGCTATATTTGCTGCACGCAAGGAAAATCTACCA  63
           ||||||||||||||||||||||||||||||||||||||||||||||||||||||||||||
Sbjct  95  CCCGAACTCAACCCGCGCCTTCGCTCTGCTATATTTGCTGCACGCAAGGAAAATCTACCA  36

Query  64  AAAGATAAAATAGAAACAGCAATAAAAAATGCAAC  98
           |||||||||||||||||||||||||||||||||||
Sbjct  35  AAAGATAAAATAGAAACAGCAATAAAAAATGCAAC  1


> 16301770 length 119 cvg_63.0_tip_0
Length=119

 Score =   172 bits (93),  Expect = 4e-41
 Identities = 93/93 (100%), Gaps = 0/93 (0%)
 Strand=Plus/Minus

Query  352  TTAGAAGTATTGAATGTTGAGGAAAATGACAAAGAAGGATTACACGTTATAACTTGTGAA  411
            ||||||||||||||||||||||||||||||||||||||||||||||||||||||||||||
Sbjct  119  TTAGAAGTATTGAATGTTGAGGAAAATGACAAAGAAGGATTACACGTTATAACTTGTGAA  60

Query  412  ATAAAAGATTTTGGTAAAGTACGCGATGCCTTT  444
            |||||||||||||||||||||||||||||||||
Sbjct  59   ATAAAAGATTTTGGTAAAGTACGCGATGCCTTT  27


> 14962368 length 103 cvg_63.0_tip_0
Length=103

 Score =   163 bits (88),  Expect = 2e-38
```

```
 Identities = 98/103 (95%), Gaps = 0/103 (0%)
 Strand=Plus/Minus

Query  312  GGGTGTGAATTTTGATGATTTATTCAGTCATGGAATCGAATTAGAAGTATTGAATGTTGA  371
            |||||||||||||||||||||||| | | |||| ||||| ||||||||||||||||||||
Sbjct  103  GGGTGTGAATTTTGATGATTTATTTAATTATGGGATCGAGTTAGAAGTATTGAATGTTGA  44

Query  372  GGAAAATGACAAAGAAGGATTACACGTTATAACTTGTGAAATA  414
            |||||||||||||||||||||||||||||||||||||||||||
Sbjct  43   GGAAAATGACAAAGAAGGATTACACGTTATAACTTGTGAAATA  1


> 13234477 length 87 cvg_61.3_tip_0
Length=87

 Score =   161 bits (87),  Expect = 9e-38
 Identities = 87/87 (100%), Gaps = 0/87 (0%)
 Strand=Plus/Plus

Query  36   ATTTGCTGCACGCAAGGAAAATCTACCAAAAGATAAAATAGAAACAGCAATAAAAAATGC  95
            ||||||||||||||||||||||||||||||||||||||||||||||||||||||||||||
Sbjct  1    ATTTGCTGCACGCAAGGAAAATCTACCAAAAGATAAAATAGAAACAGCAATAAAAAATGC  60

Query  96   AACTGGTAACGTTGCTGGAGAAAATTA  122
            |||||||||||||||||||||||||||
Sbjct  61   AACTGGTAACGTTGCTGGAGAAAATTA  87


> 11246909 length 76 cvg_63.0_tip_0
Length=76

 Score =   134 bits (72),  Expect = 2e-29
 Identities = 74/75 (99%), Gaps = 0/75 (0%)
 Strand=Plus/Plus

Query  216  ACGTTATATATTTTCTCGCAAGGGTGGAAACTTGGGAGAAACAGGAAGTGTTAGTTACCT  275
            ||||||||||||||||||||||||||||||||||||||||||||||||||||||||||||
Sbjct  1    ACGTTATATATTTTCTCGCAAGGGTGGAAACTTGGGAGAAACAGGAAGTGTTAGTTACCT  60

Query  276  TTTTGATCATGTAGG  290
            ||| |||||||||||
Sbjct  61   TTTCGATCATGTAGG  75


> 10545799 length 73 cvg_42.0_tip_0
Length=73

 Score =   122 bits (66),  Expect = 4e-26
 Identities = 66/66 (100%), Gaps = 0/66 (0%)
 Strand=Plus/Minus

Query  1   CTACCCGAACTCAACCCGCGCCTTCGCTCTGCTATATTTGCTGCACGCAAGGAAAATCTA  60
           ||||||||||||||||||||||||||||||||||||||||||||||||||||||||||||
Sbjct  66  CTACCCGAACTCAACCCGCGCCTTCGCTCTGCTATATTTGCTGCACGCAAGGAAAATCTA  7

Query  61  CCAAAA  66
           ||||||
Sbjct  6   CCAAAA  1


> 21173064 length 207 cvg_58.8_tip_0
Length=207
```

```
 Score =   117 bits (63),  Expect = 2e-24
 Identities = 63/63 (100%), Gaps = 0/63 (0%)
 Strand=Plus/Minus

Query  4    CCCGAACTCAACCCGCGCCTTCGCTCTGCTATATTTGCTGCACGCAAGGAAAATCTACCA  63
            ||||||||||||||||||||||||||||||||||||||||||||||||||||||||||||
Sbjct  63   CCCGAACTCAACCCGCGCCTTCGCTCTGCTATATTTGCTGCACGCAAGGAAAATCTACCA  4

Query  64   AAA  66
            |||
Sbjct  3    AAA  1


> 21996609 length 277 cvg_1.0_tip_1
Length=277

 Score =   115 bits (62),  Expect = 7e-24
 Identities = 62/62 (100%), Gaps = 0/62 (0%)
 Strand=Plus/Plus

Query  84   AATAAAAAATGCAACTGGTAACGTTGCTGGAGAAAATTACGAGGAAATACAATATGAAGG  143
            ||||||||||||||||||||||||||||||||||||||||||||||||||||||||||||
Sbjct  216  AATAAAAAATGCAACTGGTAACGTTGCTGGAGAAAATTACGAGGAAATACAATATGAAGG  275

Query  144  TC  145
            ||
Sbjct  276  TC  277



Lambda      K        H
    1.33    0.621     1.12

Gapped
Lambda      K        H
    1.28    0.460    0.850

Effective search space used: 426327259072
```

Query= CoxA_Wolbachia_Host_E.serratulae

```
Length=402
                                                                      Score     E
Sequences producing significant alignments:                          (Bits)  Value

  21853093 length 256 cvg_1.0_tip_1                                   414    5e-114
  20563934 length 184 cvg_38.1_tip_0                                  340    9e-92
  21022630 length 201 cvg_1.0_tip_0                                   333    2e-89
  22438024 length 412 cvg_32.5_tip_0                                  279    2e-73
  21190622 length 208 cvg_1.0_tip_1                                   272    3e-71
  20023104 length 162 cvg_62.7_tip_0                                  267    2e-69
  18361237 length 127 cvg_2.0_tip_0                                   224    1e-56
  14852056 length 102 cvg_61.1_tip_0                                  189    4e-46
  14331621 length 96 cvg_63.0_tip_0                                   178    8e-43
  15243986 length 107 cvg_63.0_tip_0                                  172    4e-41
  14097458 length 94 cvg_63.0_tip_0                                   169    5e-40
  13770563 length 91 cvg_63.0_tip_0                                   169    5e-40
  14514057 length 98 cvg_63.0_tip_0                                   159    3e-37
  13017009 length 85 cvg_63.0_tip_0                                   147    2e-33
```

```
  18281483 length 127 cvg_63.0_tip_0                                        141    1e-31
  17442278 length 126 cvg_2.0_tip_0                                         134    2e-29


> 21853093 length 256 cvg_1.0_tip_1
Length=256

 Score =   414 bits (224),  Expect = 5e-114
 Identities = 236/242 (98%), Gaps = 0/242 (0%)
 Strand=Plus/Minus

Query  1    ATGCGCACAAAAGGAATGTCATTAACTAAGATGCCACTGTTTGTTTGGTCTGTCTTGCTA  60
            ||||||||||||||||||||||||||||||||||||||||||||||||| ||||||||||
Sbjct  242  ATGCGCACAAAAGGAATGTCATTAACTAAGATGCCACTGTTTGTTTGGTATGTCTTGCTA  183

Query  61   ACAGCATTTATGTTGATTGTTGCCTTACCAGTGCTTGCCGGTGCTATAACTATGCTTCTT  120
            |||||||||||||||||||||||||||||||||||||||||||| |||||||||||||||
Sbjct  182  ACAGCATTTATGTTGATTGTTGCCTTACCAGTGCTTGCCGGTGCCATAACTATGCTTCTT  123

Query  121  ACTGATCGCAATATTGGTACTTCCTTTTTTGATCCTGCAGGTGGTGGTGACCCTGTGTTA  180
            ||||||||||||||||||||||||||||||||||||| ||||||||||||||| ||||||
Sbjct  122  ACTGATCGCAATATTGGTACTTCCTTTTTTGATCCTGTAGGTGGTGGTGACCCCGTGTTA  63

Query  181  TTTCAACATTTATTTTGGTTTTTTGGTCATCCAGAAGTTTACGTAATTATTTTTCCTGCA  240
            ||||||||| |||||||||||||||| |||||||||||||||||||||||||||||||||
Sbjct  62   TTTCAACATATATTTTGGTTTTTTGGGCATCCAGAAGTTTACGTAATTATTTTTCCTGCA  3

Query  241  TT  242
            ||
Sbjct  2    TT  1


> 20563934 length 184 cvg_38.1_tip_0
Length=184

 Score =   340 bits (184),  Expect = 9e-92
 Identities = 184/184 (100%), Gaps = 0/184 (0%)
 Strand=Plus/Minus

Query  70   ATGTTGATTGTTGCCTTACCAGTGCTTGCCGGTGCTATAACTATGCTTCTTACTGATCGC  129
            ||||||||||||||||||||||||||||||||||||||||||||||||||||||||||||
Sbjct  184  ATGTTGATTGTTGCCTTACCAGTGCTTGCCGGTGCTATAACTATGCTTCTTACTGATCGC  125

Query  130  AATATTGGTACTTCCTTTTTTGATCCTGCAGGTGGTGGTGACCCTGTGTTATTTCAACAT  189
            ||||||||||||||||||||||||||||||||||||||||||||||||||||||||||||
Sbjct  124  AATATTGGTACTTCCTTTTTTGATCCTGCAGGTGGTGGTGACCCTGTGTTATTTCAACAT  65

Query  190  TTATTTTGGTTTTTTGGTCATCCAGAAGTTTACGTAATTATTTTTCCTGCATTTGGCATC  249
            ||||||||||||||||||||||||||||||||||||||||||||||||||||||||||||
Sbjct  64   TTATTTTGGTTTTTTGGTCATCCAGAAGTTTACGTAATTATTTTTCCTGCATTTGGCATC  5

Query  250  ATAA  253
            ||||
Sbjct  4    ATAA  1


> 21022630 length 201 cvg_1.0_tip_0
Length=201

 Score =   333 bits (180),  Expect = 2e-89
 Identities = 194/201 (97%), Gaps = 0/201 (0%)
 Strand=Plus/Minus
```

```
Query  31   ATGCCACTGTTTGTTTGGTCTGTCTTGCTAACAGCATTTATGTTGATTGTTGCCTTACCA  90
            ||||||||||||||||||||||||||||||||||||||||||||||||||||||||||||
Sbjct  201  ATGCCACTGTTTGTTTGGTCTGTCTTGCTAACAGCATTTATGTTGATTGTTGCCTTACCA  142

Query  91   GTGCTTGCCGGTGCTATAACTATGCTTCTTACTGATCGCAATATTGGTACTTCCTTTTTT  150
            ||| ||||||||||||||||||||||||||||||||||| || |||||||||||||||||
Sbjct  141  GTGTTTGCCGGTGCTATAACTATGCTTCTTACTGATCGCCATGTTGGTACTTCCTTTTTT  82

Query  151  GATCCTGCAGGTGGTGGTGACCCTGTGTTATTTCAACATTTATTTGGTTTTTTGGTCAT  210
            |||||||| ||||| ||||| |||||||||||||||||| ||||||||||||||||||||
Sbjct  81   GATCCTGCCGGTGGCGGTGATCCTGTGTTATTTCAACATCTATTTGGTTTTTTGGTCAT  22

Query  211  CCAGAAGTTTACGTAATTATT  231
            |||||||||||||||||||||
Sbjct  21   CCAGAAGTTTACGTAATTATT  1


> 22438024 length 412 cvg_32.5_tip_0
Length=412

 Score =   279 bits (151),  Expect = 2e-73
 Identities = 151/151 (100%), Gaps = 0/151 (0%)
 Strand=Plus/Plus

Query  252  AAGTCAGGTTGTATCAACTTTTTCTCACAGACCTGTATTTGGTTACATAGGGATGGTTTA  311
            ||||||||||||||||||||||||||||||||||||||||||||||||||||||||||||
Sbjct  1    AAGTCAGGTTGTATCAACTTTTTCTCACAGACCTGTATTTGGTTACATAGGGATGGTTTA  60

Query  312  TGCAATGATAGGTATAGCAGTATTTGGCTTTATGGTTTGGGCTCACCATATGTTCACTGT  371
            ||||||||||||||||||||||||||||||||||||||||||||||||||||||||||||
Sbjct  61   TGCAATGATAGGTATAGCAGTATTTGGCTTTATGGTTTGGGCTCACCATATGTTCACTGT  120

Query  372  TGGGCTTAGTGCTGACGCTGCTGCATTTTTT  402
            |||||||||||||||||||||||||||||||
Sbjct  121  TGGGCTTAGTGCTGACGCTGCTGCATTTTTT  151


> 21190622 length 208 cvg_1.0_tip_1
Length=208

 Score =   272 bits (147),  Expect = 3e-71
 Identities = 157/162 (97%), Gaps = 0/162 (0%)
 Strand=Plus/Minus

Query  1    ATGCGCACAAAAGGAATGTCATTAACTAAGATGCCACTGTTTGTTTGGTCTGTCTTGCTA  60
            ||||||||||||||||| ||||||||||||||| ||||||||||||||| ||||||||||
Sbjct  162  ATGCGCACAAAAGGAATATCATTAACTAAGATGACACTGTTTGTTTGGTATGTCTTGCTA  103

Query  61   ACAGCATTTATGTTGATTGTTGCCTTACCAGTGCTTGCCGGTGCTATAACTATGCTTCTT  120
            |||||||||||||||||| |||||||||||||||||||||||||||||||||||||||||
Sbjct  102  ACAGCATTTATGTTGATTATTGCCTTACCAGTGCTTGCCGGTGCTATAACTATGCTTCTT  43

Query  121  ACTGATCGCAATATTGGTACTTCCTTTTTTGATCCTGCAGGT  162
            |||||||| |||||||||||||||||||||||||||||||||
Sbjct  42   ACTGATCGAAATATTGGTACTTCCTTTTTTGATCCTGCAGGT  1


> 20023104 length 162 cvg_62.7_tip_0
Length=162

 Score =   267 bits (144),  Expect = 2e-69
 Identities = 156/162 (96%), Gaps = 0/162 (0%)
 Strand=Plus/Minus
```

```
Query  70   ATGTTGATTGTTGCCTTACCAGTGCTTGCCGGTGCTATAACTATGCTTCTTACTGATCGC  129
            ||||||||||||||||||||||||||||||||||||||||||||||||||||||||||||
Sbjct  162  ATGTTGATTGTTGCCTTACCAGTGCTTGCCGGTGCTATAACTATGCTTCTTACTGATCGC  103

Query  130  AATATTGGTACTTCCTTTTTTGATCCTGCAGGTGGTGGTGACCCTGTGTTATTTCAACAT  189
            ||| ||||||||||||||||||||||||| ||||| || || ||||||||||||||||||
Sbjct  102  AATGTTGGTACTTCCTTTTTTGATCCTGCCGGTGGCGGAGATCCTGTGTTATTTCAACAT  43

Query  190  TTATTTTGGTTTTTTGGTCATCCAGAAGTTTACGTAATTATT  231
             |||||||||||||||||||||||||||||||||||||||||
Sbjct  42   CTATTTTGGTTTTTTGGTCATCCAGAAGTTTACGTAATTATT  1


> 18361237 length 127 cvg_2.0_tip_0
Length=127

 Score =   224 bits (121),  Expect = 1e-56
 Identities = 125/127 (98%), Gaps = 0/127 (0%)
 Strand=Plus/Minus


Query  219  TTACGTAATTATTTTTCCTGCATTTGGCATCATAAGTCAGGTTGTATCAACTTTTTCTCA  278
            ||||||||||||||||||||||||||||||||||||||||||||||||||||||||||||
Sbjct  127  TTACGTAATTATTTTTCCTGCATTTGGCATCATAAGTCAGGTTGTATCAACTTTTTCTCA  68

Query  279  CAGACCTGTATTTGGTTACATAGGGATGGTTTATGCAATGATAGGTATAGCAGTATTTGG  338
            ||| |||||||||||||||||||||||||||||||| |||||||||||||||||||||||
Sbjct  67   CAGGCCTGTATTTGGTTACATAGGGATGGTTTATGCTATGATAGGTATAGCAGTATTTGG  8

Query  339  CTTTATG  345
            |||||||
Sbjct  7    CTTTATG  1


> 14852056 length 102 cvg_61.1_tip_0
Length=102

 Score =   189 bits (102),  Expect = 4e-46
 Identities = 102/102 (100%), Gaps = 0/102 (0%)
 Strand=Plus/Minus

Query  31   ATGCCACTGTTTGTTTGGTCTGTCTTGCTAACAGCATTTATGTTGATTGTTGCCTTACCA  90
            ||||||||||||||||||||||||||||||||||||||||||||||||||||||||||||
Sbjct  102  ATGCCACTGTTTGTTTGGTCTGTCTTGCTAACAGCATTTATGTTGATTGTTGCCTTACCA  43

Query  91   GTGCTTGCCGGTGCTATAACTATGCTTCTTACTGATCGCAAT  132
            ||||||||||||||||||||||||||||||||||||||||||
Sbjct  42   GTGCTTGCCGGTGCTATAACTATGCTTCTTACTGATCGCAAT  1


> 14331621 length 96 cvg_63.0_tip_0
Length=96

 Score =   178 bits (96),  Expect = 8e-43
 Identities = 96/96 (100%), Gaps = 0/96 (0%)
 Strand=Plus/Minus

Query  219  TTACGTAATTATTTTTCCTGCATTTGGCATCATAAGTCAGGTTGTATCAACTTTTTCTCA  278
            ||||||||||||||||||||||||||||||||||||||||||||||||||||||||||||
Sbjct  96   TTACGTAATTATTTTTCCTGCATTTGGCATCATAAGTCAGGTTGTATCAACTTTTTCTCA  37

Query  279  CAGACCTGTATTTGGTTACATAGGGATGGTTTATGC  314
```

```
            ||||||||||||||||||||||||||||||||||||
Sbjct  36   CAGACCTGTATTTGGTTACATAGGGATGGTTTATGC  1


> 15243986 length 107 cvg_63.0_tip_0
Length=107

 Score =   172 bits (93),  Expect = 4e-41
 Identities = 93/93 (100%), Gaps = 0/93 (0%)
 Strand=Plus/Plus

Query  1    ATGCGCACAAAAGGAATGTCATTAACTAAGATGCCACTGTTTGTTTGGTCTGTCTTGCTA  60
            ||||||||||||||||||||||||||||||||||||||||||||||||||||||||||||
Sbjct  15   ATGCGCACAAAAGGAATGTCATTAACTAAGATGCCACTGTTTGTTTGGTCTGTCTTGCTA  74

Query  61   ACAGCATTTATGTTGATTGTTGCCTTACCAGTG  93
            |||||||||||||||||||||||||||||||||
Sbjct  75   ACAGCATTTATGTTGATTGTTGCCTTACCAGTG  107


> 14097458 length 94 cvg_63.0_tip_0
Length=94

 Score =   169 bits (91),  Expect = 5e-40
 Identities = 93/94 (99%), Gaps = 0/94 (0%)
 Strand=Plus/Plus

Query  252  AAGTCAGGTTGTATCAACTTTTTCTCACAGACCTGTATTTGGTTACATAGGGATGGTTTA  311
            ||||||||||||||||||||||||||||||||||||||||||||||||||||||||||||
Sbjct  1    AAGTCAGGTTGTATCAACTTTTTCTCACAGACCTGTATTTGGTTACATAGGGATGGTTTA  60

Query  312  TGCAATGATAGGTATAGCAGTATTTGGCTTTATG  345
            ||| ||||||||||||||||||||||||||||||
Sbjct  61   TGCTATGATAGGTATAGCAGTATTTGGCTTTATG  94


> 13770563 length 91 cvg_63.0_tip_0
Length=91

 Score =   169 bits (91),  Expect = 5e-40
 Identities = 91/91 (100%), Gaps = 0/91 (0%)
 Strand=Plus/Minus

Query  191  TATTTTGGTTTTTTGGTCATCCAGAAGTTTACGTAATTATTTTTCCTGCATTTGGCATCA  250
            ||||||||||||||||||||||||||||||||||||||||||||||||||||||||||||
Sbjct  91   TATTTTGGTTTTTTGGTCATCCAGAAGTTTACGTAATTATTTTTCCTGCATTTGGCATCA  32

Query  251  TAAGTCAGGTTGTATCAACTTTTTCTCACAG  281
            |||||||||||||||||||||||||||||||
Sbjct  31   TAAGTCAGGTTGTATCAACTTTTTCTCACAG  1


> 14514057 length 98 cvg_63.0_tip_0
Length=98

 Score =   159 bits (86),  Expect = 3e-37
 Identities = 94/98 (96%), Gaps = 0/98 (0%)
 Strand=Plus/Minus

Query  283  CCTGTATTTGGTTACATAGGGATGGTTTATGCAATGATAGGTATAGCAGTATTTGGCTTT  342
            |||||||||||||||||||||||||||||||| |||||||||||||||||||||||||||
Sbjct  98   CCTGTATTTGGTTACATAGGGATGGTTTATGCTATGATAGGTATAGCAGTATTTGGCTTT  39
```

```
Query  343   ATGGTTTGGGCTCACCATATGTTCACTGTTGGGCTTAG  380
             ||||| |||| ||| ||||||||||||||||||||||
Sbjct  38    ATGGTGTGGGTTCATCATATGTTCACTGTTGGGCTTAG  1


> 13017009 length 85 cvg_63.0_tip_0
Length=85

 Score =   147 bits (79),  Expect = 2e-33
 Identities = 81/82 (99%), Gaps = 0/82 (0%)
 Strand=Plus/Minus

Query  172   CCTGTGTTATTTCAACATTTATTTTGGTTTTTTGGTCATCCAGAAGTTTACGTAATTATT  231
             ||||||||||||||||| ||||||||||||||||||||||||||||||||||||||||||
Sbjct  82    CCTGTGTTATTTCAACATCTATTTTGGTTTTTTGGTCATCCAGAAGTTTACGTAATTATT  23

Query  232   TTTCCTGCATTTGGCATCATAA  253
             ||||||||||||||||||||||
Sbjct  22    TTTCCTGCATTTGGCATCATAA  1


> 18281483 length 127 cvg_63.0_tip_0
Length=127

 Score =   141 bits (76),  Expect = 1e-31
 Identities = 82/85 (96%), Gaps = 0/85 (0%)
 Strand=Plus/Plus

Query  318   GATAGGTATAGCAGTATTTGGCTTTATGGTTTGGGCTCACCATATGTTCACTGTTGGGCT  377
             ||||||||||||||||||||||||||||| |||| ||| ||||||||||||||||||||
Sbjct  1     GATAGGTATAGCAGTATTTGGCTTTATGGTGTGGGTTCATCATATGTTCACTGTTGGGCT  60

Query  378   TAGTGCTGACGCTGCTGCATTTTTT  402
             |||||||||||||||||||||||||
Sbjct  61    TAGTGCTGACGCTGCTGCATTTTTT  85


> 17442278 length 126 cvg_2.0_tip_0
Length=126

 Score =   134 bits (72),  Expect = 2e-29
 Identities = 81/85 (95%), Gaps = 1/85 (1%)
 Strand=Plus/Minus

Query  318   GATAGGTATAGCAGTATTTGGCTTTATGGTTTGGGCTCACCATATGTTCACTGTTGGGCT  377
             ||||||||||||||||||||||||||||| |||| ||| ||||||||||||||||||||
Sbjct  126   GATAGGTATAGCAGTATTTGGCTTTATGGTGTGGGTTCATCATATGTTCACTGTTGGGCT  67

Query  378   TAGTGCTGACGCTGCTGCATTTTTT  402
             ||| |||||||||||||||||||||
Sbjct  66    TAG-GCTGACGCTGCTGCATTTTTT  43



Lambda      K        H
    1.33    0.621     1.12

Gapped
Lambda      K        H
    1.28    0.460    0.850

Effective search space used: 383284603108
```

Query= fstZ_Wolbachia_Host_E.serratulae

```
Length=435
                                                                          Score     E
Sequences producing significant alignments:                              (Bits)  Value

  20372568 length 175 cvg_39.8_tip_0                                        324   1e-86
  20373274 length 175 cvg_62.6_tip_0                                        313   2e-83
  18459913 length 127 cvg_41.4_tip_0                                        235   5e-60
  21442191 length 221 cvg_1.0_tip_1                                         230   2e-58
  22135616 length 301 cvg_38.6_tip_0                                        228   8e-58
  17614347 length 127 cvg_2.0_tip_0                                         224   1e-56
  20450530 length 179 cvg_63.0_tip_0                                        217   2e-54
  15315059 length 108 cvg_63.0_tip_0                                        200   2e-49
  21144322 length 206 cvg_1.0_tip_0                                         193   3e-47
  14485123 length 98 cvg_63.0_tip_0                                         182   6e-44
  14246929 length 95 cvg_61.9_tip_0                                         176   3e-42
  14373293 length 97 cvg_63.0_tip_0                                         174   1e-41
  13200020 length 86 cvg_63.0_tip_0                                         159   3e-37
  12489093 length 82 cvg_63.0_tip_0                                         147   2e-33
  10590477 length 74 cvg_63.0_tip_0                                         132   7e-29
  19289967 length 138 cvg_2.0_tip_0                                         128   8e-28
  14421785 length 97 cvg_63.0_tip_0                                         119   5e-25
  20052480 length 163 cvg_55.0_tip_0                                        117   2e-24
  21412982 length 219 cvg_1.0_tip_1                                         108   1e-21


> 20372568 length 175 cvg_39.8_tip_0
Length=175

 Score =   324 bits (175),  Expect = 1e-86
 Identities = 175/175 (100%), Gaps = 0/175 (0%)
 Strand=Plus/Plus

Query  228  TAGAATTGCAAATGAAAAAACTACATTTTCTGATGCATTTAAACTTGCTGATAATGTTCT  287
            ||||||||||||||||||||||||||||||||||||||||||||||||||||||||||||
Sbjct  1    TAGAATTGCAAATGAAAAAACTACATTTTCTGATGCATTTAAACTTGCTGATAATGTTCT  60

Query  288  GCACATTGGCATCAGAGGAGTAACTGACTTGATGGTCATGCCAGGGCTTATCAATCTTGA  347
            ||||||||||||||||||||||||||||||||||||||||||||||||||||||||||||
Sbjct  61   GCACATTGGCATCAGAGGAGTAACTGACTTGATGGTCATGCCAGGGCTTATCAATCTTGA  120

Query  348  CTTCGCTGATATAGAAACAGTAATGAGCGAGATGGGCAAAGCGATGATCGGCACC  402
            |||||||||||||||||||||||||||||||||||||||||||||||||||||||
Sbjct  121  CTTCGCTGATATAGAAACAGTAATGAGCGAGATGGGCAAAGCGATGATCGGCACC  175


> 20373274 length 175 cvg_62.6_tip_0
Length=175

 Score =   313 bits (169),  Expect = 2e-83
 Identities = 173/175 (99%), Gaps = 0/175 (0%)
 Strand=Plus/Plus

Query  228  TAGAATTGCAAATGAAAAAACTACATTTTCTGATGCATTTAAACTTGCTGATAATGTTCT  287
            ||||||||||||||||||||||||||||||||||||||||||||||||||||||||||||
Sbjct  1    TAGAATTGCAAATGAAAAAACTACATTTTCTGATGCATTTAAACTTGCTGATAATGTTCT  60

Query  288  GCACATTGGCATCAGAGGAGTAACTGACTTGATGGTCATGCCAGGGCTTATCAATCTTGA  347
```

```
              ||| ||||||||||||||||||||||||||||||||||||||||||||||| ||||||||
Sbjct  61     GCATATTGGCATCAGAGGAGTAACTGACTTGATGGTCATGCCAGGGCTTATTAATCTTGA  120

Query  348    CTTCGCTGATATAGAAACAGTAATGAGCGAGATGGGCAAAGCGATGATCGGCACC  402
              |||||||||||||||||||||||||||||||||||||||||||||||||||||||
Sbjct  121    CTTCGCTGATATAGAAACAGTAATGAGCGAGATGGGCAAAGCGATGATCGGCACC  175


> 18459913 length 127 cvg_41.4_tip_0
Length=127

 Score =   235 bits (127),  Expect = 5e-60
 Identities = 127/127 (100%), Gaps = 0/127 (0%)
 Strand=Plus/Plus

Query  84     AGaaaaaaaGATATTGACTGTTGGAGTTGTAACTAAACCGTTCGGTTTTGAAGGTGTGCG  143
              ||||||||||||||||||||||||||||||||||||||||||||||||||||||||||||
Sbjct  1      AGAAAAAAAGATATTGACTGTTGGAGTTGTAACTAAACCGTTCGGTTTTGAAGGTGTGCG  60

Query  144    CCGTATGCGCATTGCAGAGCTTGGACTTGAAGAACTGCAAAAATACGTGGATACACTTAT  203
              ||||||||||||||||||||||||||||||||||||||||||||||||||||||||||||
Sbjct  61     CCGTATGCGCATTGCAGAGCTTGGACTTGAAGAACTGCAAAAATACGTGGATACACTTAT  120

Query  204    TGTCATT  210
              |||||||
Sbjct  121    TGTCATT  127


> 21442191 length 221 cvg_1.0_tip_1
Length=221

 Score =   230 bits (124),  Expect = 2e-58
 Identities = 134/139 (96%), Gaps = 0/139 (0%)
 Strand=Plus/Plus

Query  297    CATCAGAGGAGTAACTGACTTGATGGTCATGCCAGGGCTTATCAATCTTGACTTCGCTGA  356
              |||||||||||||||||||||||||| || |||||||||||| |||||||||||||||||
Sbjct  1      CATCAGAGGAGTAACTGACTTGATGGGCAGGCCAGGGCTTATTAATCTTGACTTCGCTGA  60

Query  357    TATAGAAACAGTAATGAGCGAGATGGGCAAAGCGATGATCGGCACCGGAGAGGCAGAAGG  416
              || ||||||||||||||||||||||||||||||||||||| |||||||||||||||||||
Sbjct  61     TAGAGAAACAGTAATGAGCGAGATGGGCAAAGCGATGATCTGCACCGGAGAGGCAGAAGG  120

Query  417    AGAAGATAGAGCAATTAGT  435
              |||||||||||||||||||
Sbjct  121    AGAAGATAGAGCAATTAGT  139


> 22135616 length 301 cvg_38.6_tip_0
Length=301

 Score =   228 bits (123),  Expect = 8e-58
 Identities = 123/123 (100%), Gaps = 0/123 (0%)
 Strand=Plus/Plus

Query  1      GGTGGTACTGGAACCGGTGCAGCACCGGTAATTGCAAAAGCAGCCAGAGAAGCAAGAGCC  60
              ||||||||||||||||||||||||||||||||||||||||||||||||||||||||||||
Sbjct  179    GGTGGTACTGGAACCGGTGCAGCACCGGTAATTGCAAAAGCAGCCAGAGAAGCAAGAGCC  238

Query  61     GCAGTTAAGGATAGAGCGCCAAAAGaaaaaaaGATATTGACTGTTGGAGTTGTAACTAAA  120
              ||||||||||||||||||||||||||||||||||||||||||||||||||||||||||||
Sbjct  239    GCAGTTAAGGATAGAGCGCCAAAAGAAAAAAAGATATTGACTGTTGGAGTTGTAACTAAA  298
```

```
Query  121  CCG  123
            |||
Sbjct  299  CCG  301


> 17614347 length 127 cvg_2.0_tip_0
Length=127

 Score =   224 bits (121),  Expect = 1e-56
 Identities = 125/127 (98%), Gaps = 0/127 (0%)
 Strand=Plus/Minus

Query  129  TTTTGAAGGTGTGCGCCGTATGCGCATTGCAGAGCTTGGACTTGAAGAACTGCAAAAATA  188
            |||||||||||||||||| |||||||||||||||||||||||||||||||||||||||||
Sbjct  127  TTTTGAAGGTGTGCGCCGCATGCGCATTGCAGAGCTTGGACTTGAAGAACTGCAAAAATA  68

Query  189  CGTGGATACACTTATTGTCATTCCAAATCAGAATTTATTTAGAATTGCAAATGAAAAAAC  248
            ||| ||||||||||||||||||||||||||||||||||||||||||||||||||||||||
Sbjct  67   CGTTGATACACTTATTGTCATTCCAAATCAGAATTTATTTAGAATTGCAAATGAAAAAAC  8

Query  249  TACATTT  255
            |||||||
Sbjct  7    TACATTT  1


> 20450530 length 179 cvg_63.0_tip_0
Length=179

 Score =   217 bits (117),  Expect = 2e-54
 Identities = 121/123 (98%), Gaps = 0/123 (0%)
 Strand=Plus/Minus

Query  1    GGTGGTACTGGAACCGGTGCAGCACCGGTAATTGCAAAAGCAGCCAGAGAAGCAAGAGCC  60
            |||||||| |||||||||||||||||||||||||||||||||||||||||||||||||||
Sbjct  123  GGTGGTACCGGAACCGGTGCAGCACCGGTAATTGCAAAAGCAGCCAGAGAAGCAAGAGCT  64

Query  61   GCAGTTAAGGATAGAGCGCCAAAAGaaaaaaaGATATTGACTGTTGGAGTTGTAACTAAA  120
            ||||||||||||||||||||||||||||||||||||||||||||||||||||||||||||
Sbjct  63   GCAGTTAAGGATAGAGCGCCAAAAGAAAAAAAGATATTGACTGTTGGAGTTGTAACTAAA  4

Query  121  CCG  123
            |||
Sbjct  3    CCG  1


> 15315059 length 108 cvg_63.0_tip_0
Length=108

 Score =   200 bits (108),  Expect = 2e-49
 Identities = 108/108 (100%), Gaps = 0/108 (0%)
 Strand=Plus/Plus

Query  148  ATGCGCATTGCAGAGCTTGGACTTGAAGAACTGCAAAAATACGTGGATACACTTATTGTC  207
            ||||||||||||||||||||||||||||||||||||||||||||||||||||||||||||
Sbjct  1    ATGCGCATTGCAGAGCTTGGACTTGAAGAACTGCAAAAATACGTGGATACACTTATTGTC  60

Query  208  ATTCCAAATCAGAATTTATTTAGAATTGCAAATGAAAAAACTACATTT  255
            ||||||||||||||||||||||||||||||||||||||||||||||||
Sbjct  61   ATTCCAAATCAGAATTTATTTAGAATTGCAAATGAAAAAACTACATTT  108


> 21144322 length 206 cvg_1.0_tip_0
Length=206
```

```
 Score =   193 bits (104),  Expect = 3e-47
 Identities = 110/113 (97%), Gaps = 0/113 (0%)
 Strand=Plus/Minus

Query  323  TCATGCCAGGGCTTATCAATCTTGACTTCGCTGATATAGAAACAGTAATGAGCGAGATGG  382
            |||||||||||||||| |||||||||||||||||||| ||||||||||||||||||||||
Sbjct  146  TCATGCCAGGGCTTATTAATCTTGACTTCGCTGATATGGAAACAGTAATGAGCGAGATGG  87

Query  383  GCAAAGCGATGATCGGCACCGGAGAGGCAGAAGGAGAAGATAGAGCAATTAGT  435
            |||||||||||||||||||||| ||||||||||||||||||||||||||||||
Sbjct  86   GCAAAGCGATGATCGGCACCGGGGAGGCAGAAGGAGAAGATAGAGCAATTAGT  34


> 14485123 length 98 cvg_63.0_tip_0
Length=98

 Score =   182 bits (98),  Expect = 6e-44
 Identities = 98/98 (100%), Gaps = 0/98 (0%)
 Strand=Plus/Minus

Query  193  GATACACTTATTGTCATTCCAAATCAGAATTTATTTAGAATTGCAAATGAAAAAACTACA  252
            ||||||||||||||||||||||||||||||||||||||||||||||||||||||||||||
Sbjct  98   GATACACTTATTGTCATTCCAAATCAGAATTTATTTAGAATTGCAAATGAAAAAACTACA  39

Query  253  TTTTCTGATGCATTTAAACTTGCTGATAATGTTCTGCA  290
            ||||||||||||||||||||||||||||||||||||||
Sbjct  38   TTTTCTGATGCATTTAAACTTGCTGATAATGTTCTGCA  1


> 14246929 length 95 cvg_61.9_tip_0
Length=95

 Score =   176 bits (95),  Expect = 3e-42
 Identities = 95/95 (100%), Gaps = 0/95 (0%)
 Strand=Plus/Minus

Query  340  AATCTTGACTTCGCTGATATAGAAACAGTAATGAGCGAGATGGGCAAAGCGATGATCGGC  399
            ||||||||||||||||||||||||||||||||||||||||||||||||||||||||||||
Sbjct  95   AATCTTGACTTCGCTGATATAGAAACAGTAATGAGCGAGATGGGCAAAGCGATGATCGGC  36

Query  400  ACCGGAGAGGCAGAAGGAGAAGATAGAGCAATTAG  434
            |||||||||||||||||||||||||||||||||||
Sbjct  35   ACCGGAGAGGCAGAAGGAGAAGATAGAGCAATTAG  1


> 14373293 length 97 cvg_63.0_tip_0
Length=97

 Score =   174 bits (94),  Expect = 1e-41
 Identities = 96/97 (99%), Gaps = 0/97 (0%)
 Strand=Plus/Plus

Query  84   AGaaaaaaaGATATTGACTGTTGGAGTTGTAACTAAACCGTTCGGTTTTGAAGGTGTGCG  143
            ||||||||||||||||||||||||||||||||||||||||||||||||||||||||||||
Sbjct  1    AGAAAAAAAGATATTGACTGTTGGAGTTGTAACTAAACCGTTCGGTTTTGAAGGTGTGCG  60

Query  144  CCGTATGCGCATTGCAGAGCTTGGACTTGAAGAACTG  180
            ||| |||||||||||||||||||||||||||||||||
Sbjct  61   CCGCATGCGCATTGCAGAGCTTGGACTTGAAGAACTG  97


> 13200020 length 86 cvg_63.0_tip_0
```

```
Length=86

 Score =   159 bits (86),  Expect = 3e-37
 Identities = 86/86 (100%), Gaps = 0/86 (0%)
 Strand=Plus/Minus

Query  61   GCAGTTAAGGATAGAGCGCCAAAAGaaaaaaaGATATTGACTGTTGGAGTTGTAACTAAA  120
            ||||||||||||||||||||||||||||||||||||||||||||||||||||||||||||
Sbjct  86   GCAGTTAAGGATAGAGCGCCAAAAGAAAAAAAGATATTGACTGTTGGAGTTGTAACTAAA  27

Query  121  CCGTTCGGTTTTGAAGGTGTGCGCCG  146
            ||||||||||||||||||||||||||
Sbjct  26   CCGTTCGGTTTTGAAGGTGTGCGCCG  1


> 12489093 length 82 cvg_63.0_tip_0
Length=82

 Score =   147 bits (79),  Expect = 2e-33
 Identities = 81/82 (99%), Gaps = 0/82 (0%)
 Strand=Plus/Minus

Query  129  TTTTGAAGGTGTGCGCCGTATGCGCATTGCAGAGCTTGGACTTGAAGAACTGCAAAAATA  188
            |||||||||||||||||| |||||||||||||||||||||||||||||||||||||||||
Sbjct  82   TTTTGAAGGTGTGCGCCGCATGCGCATTGCAGAGCTTGGACTTGAAGAACTGCAAAAATA  23

Query  189  CGTGGATACACTTATTGTCATT  210
            ||||||||||||||||||||||
Sbjct  22   CGTGGATACACTTATTGTCATT  1


> 10590477 length 74 cvg_63.0_tip_0
Length=74

 Score =   132 bits (71),  Expect = 7e-29
 Identities = 73/74 (99%), Gaps = 0/74 (0%)
 Strand=Plus/Plus

Query  118  AAACCGTTCGGTTTTGAAGGTGTGCGCCGTATGCGCATTGCAGAGCTTGGACTTGAAGAA  177
            ||||||||||||||||||||||||||||| ||||||||||||||||||||||||||||||
Sbjct  1    AAACCGTTCGGTTTTGAAGGTGTGCGCCGCATGCGCATTGCAGAGCTTGGACTTGAAGAA  60

Query  178  CTGCAAAAATACGT  191
            ||||||||||||||
Sbjct  61   CTGCAAAAATACGT  74


> 19289967 length 138 cvg_2.0_tip_0
Length=138

 Score =   128 bits (69),  Expect = 8e-28
 Identities = 74/76 (97%), Gaps = 1/76 (1%)
 Strand=Plus/Plus

Query  118  AAACCGTTCGGTTTTGAAGGTGTGCGCCGTATGCGCATTGCAGAGCTTGGACTTGAAGAA  177
            ||||||||||||||||||||||||||||| ||||||||||||||||||||||||||||||
Sbjct  1    AAACCGTTCGGTTTTGAAGGTGTGCGCCGCATGCGCATTGCAGAGCTTGGACTTGAAGAA  60

Query  178  CTGCAAAAATACGTGG  193
            ||| ||||||||||||
Sbjct  61   CTG-AAAAATACGTGG  75
```

```
> 14421785 length 97 cvg_63.0_tip_0
Length=97

 Score =   119 bits (64),  Expect = 5e-25
 Identities = 64/64 (100%), Gaps = 0/64 (0%)
 Strand=Plus/Minus

Query  372   GAGCGAGATGGGCAAAGCGATGATCGGCACCGGAGAGGCAGAAGGAGAAGATAGAGCAAT  431
             ||||||||||||||||||||||||||||||||||||||||||||||||||||||||||||
Sbjct  97    GAGCGAGATGGGCAAAGCGATGATCGGCACCGGAGAGGCAGAAGGAGAAGATAGAGCAAT  38

Query  432   TAGT  435
             ||||
Sbjct  37    TAGT  34


> 20052480 length 163 cvg_55.0_tip_0
Length=163

 Score =   117 bits (63),  Expect = 2e-24
 Identities = 63/63 (100%), Gaps = 0/63 (0%)
 Strand=Plus/Minus

Query  372   GAGCGAGATGGGCAAAGCGATGATCGGCACCGGAGAGGCAGAAGGAGAAGATAGAGCAAT  431
             ||||||||||||||||||||||||||||||||||||||||||||||||||||||||||||
Sbjct  163   GAGCGAGATGGGCAAAGCGATGATCGGCACCGGAGAGGCAGAAGGAGAAGATAGAGCAAT  104

Query  432   TAG  434
             |||
Sbjct  103   TAG  101


> 21412982 length 219 cvg_1.0_tip_1
Length=219

 Score =   108 bits (58),  Expect = 1e-21
 Identities = 60/61 (98%), Gaps = 0/61 (0%)
 Strand=Plus/Plus

Query  229   AGAATTGCAAATGAAAAAACTACATTTTCTGATGCATTTAAACTTGCTGATAATGTTCTG  288
             ||||||| ||||||||||||||||||||||||||||||||||||||||||||||||||||
Sbjct  159   AGAATTGAAAATGAAAAAACTACATTTTCTGATGCATTTAAACTTGCTGATAATGTTCTG  218

Query  289   C  289
             |
Sbjct  219   C  219



Lambda      K        H
    1.33    0.621     1.12

Gapped
Lambda      K        H
    1.28    0.460    0.850

Effective search space used: 417103832794
```

Query= gatB_Wolbachia_Host_E.serratulae

```
Length=369
                                                                      Score     E
Sequences producing significant alignments:                          (Bits)  Value

  21381538 length 218 cvg_1.0_tip_1                                    357    8e-97
  20417994 length 177 cvg_2.0_tip_0                                    316    1e-84
  20741170 length 191 cvg_1.0_tip_1                                    305    3e-81
  20109650 length 165 cvg_63.0_tip_0                                   294    7e-78
  19292641 length 138 cvg_33.4_tip_0                                   255    3e-66
  18969591 length 130 cvg_63.0_tip_0                                   230    2e-58
  18736645 length 127 cvg_2.0_tip_0                                    230    2e-58
  18076019 length 127 cvg_63.0_tip_0                                   211    7e-53
  14638785 length 99 cvg_47.0_tip_0                                    183    1e-44
  15078638 length 105 cvg_46.9_tip_0                                   176    2e-42
  14245495 length 95 cvg_63.0_tip_0                                    176    2e-42
  21871023 length 258 cvg_1.0_tip_1                                    171    1e-40
  13582182 length 89 cvg_32.4_tip_0                                    165    5e-39
  13402912 length 88 cvg_49.6_tip_0                                    163    2e-38
  13056619 length 85 cvg_44.0_tip_0                                    158    9e-37
  13056441 length 85 cvg_63.0_tip_0                                    158    9e-37
  21564999 length 229 cvg_37.0_tip_0                                   135    4e-30
  10262355 length 73 cvg_63.0_tip_0                                    135    4e-30
  10213031 length 72 cvg_63.0_tip_0                                    134    2e-29
  8722663 length 69 cvg_47.0_tip_0                                     128    7e-28
  20838530 length 194 cvg_62.4_tip_0                                   124    9e-27
  3629341 length 64 cvg_0.0_tip_0                                      119    4e-25


> 21381538 length 218 cvg_1.0_tip_1
Length=218
```

Not in Wolbachia MLST database, Blast -> most related Drosophila ananassae sequence. But this latter sequence is identical to Wol-allele 22

```
 Score =   357 bits (193),  Expect = 8e-97
 Identities = 209/217 (96%), Gaps = 0/217 (0%)
 Strand=Plus/Plus

Query  153   AAATCTGAACTCGATACGTTATATTGTGCAAGCTATAGACTATGAAATACAAAGACAAAT  212
             ||||||||||||||||||||||||||||||||||||||||||||||||||||||||||||
Sbjct  1     AAATCTGAACTCGATACGTTATATTGTGCAAGCTATAGACTATGAAATACAAAGACAAAT  60

Query  213   TGAAATTTTAGAAAGTGGGGAAGAAATAAGTCAAGATACCTTATTGTTTGATGTTGCTTC  272
             ||| |||||||||||||||||||||||||||||  |||||||||||||||||||||||||
Sbjct  61    TGACATTTTAGAAAGTGGGGAAGAAATAAGTCACTATACCTTATTGTTTGATGTTGCTTC  120

Query  273   GGGAAAAACAAAAGTGATGCGAAACAAAGAAGATGCAAGCGACTATAGATACTTCCCTGA  332
             |||||| |||| ||||||||||||||||||||||||||||| ||| ||||||||||||||
Sbjct  121   GGGAAACACAACAGTGATGCGAAACAAAGAAGATGCAAGCGCCTACAGATACTTCCCTGA  180

Query  333   GCCTGATTTATTACCTGTTGAGGTAAGGCAGGATAAA  369
             ||| |||||||||||||||||||||||||||||||||
Sbjct  181   GCCCGATTTATTACCTGTTGAGGTAAGGCAGGATAAA  217


> 20417994 length 177 cvg_2.0_tip_0
Length=177

 Score =   316 bits (171),  Expect = 1e-84
 Identities = 175/177 (99%), Gaps = 0/177 (0%)
```

```
 Strand=Plus/Minus

Query  125   GCGCACTTGGCACTCGTTGTGAGATAAAAAATCTGAACTCGATACGTTATATTGTGCAAG  184
             ||||||||||||||||||||||||||||||||||||||||||||||||||||||||||||
Sbjct  177   GCGCACTTGGCACTCGTTGTGAGATAAAAAATCTGAACTCGATACGTTATATTGTGCAAG  118

Query  185   CTATAGACTATGAAATACAAAGACAAATTGAAATTTTAGAAAGTGGGGAAGAAATAAGTC  244
             ||| ||||||||||||||||||||||||||||||||||||||||||||||||| ||||||
Sbjct  117   CTAAAGACTATGAAATACAAAGACAAATTGAAATTTTAGAAAGTGGGGAAGAAGTAAGTC  58

Query  245   AAGATACCTTATTGTTTGATGTTGCTTCGGGAAAAACAAAAGTGATGCGAAACAAAG  301
             |||||||||||||||||||||||||||||||||||||||||||||||||||||||||
Sbjct  57    AAGATACCTTATTGTTTGATGTTGCTTCGGGAAAAACAAAAGTGATGCGAAACAAAG  1


> 20741170 length 191 cvg_1.0_tip_1
Length=191

 Score =   305 bits (165),  Expect = 3e-81
 Identities = 171/174 (98%), Gaps = 0/174 (0%)
 Strand=Plus/Minus

Query  1     GAAGCTGCAGAATGCATGAAAAAATTGAGGCAGATTTTGCGTTACATTGGTTCGTGTGAT  60
             |||||||||||||||||||||||||||||||||||||||||||||| |||||||||||||
Sbjct  174   GAAGCTGCAGAATGCATGAAAAAATTGAGGCAGATTTTGCGTTACAGTGGTTCGTGTGAT  115

Query  61    GGTGATATGGAAAAGGGATCACTTCGTTGTGATGCAAATGTTTCTGTCCGCCTAAAAGGC  120
             |||||||||||||||||||||| ||||||||||||||||||||||||||| |||||||||
Sbjct  114   GGTGATATGGAAAAGGGATCACGTCGTTGTGATGCAAATGTTTCTGTCCGACTAAAAGGC  55

Query  121   AGTAGCGCACTTGGCACTCGTTGTGAGATAAAAAATCTGAACTCGATACGTTAT  174
             ||||||||||||||||||||||||||||||||||||||||||||||||||||||
Sbjct  54    AGTAGCGCACTTGGCACTCGTTGTGAGATAAAAAATCTGAACTCGATACGTTAT  1


> 20109650 length 165 cvg_63.0_tip_0
Length=165

 Score =   294 bits (159),  Expect = 7e-78
 Identities = 163/165 (99%), Gaps = 0/165 (0%)
 Strand=Plus/Minus

Query  163   TCGATACGTTATATTGTGCAAGCTATAGACTATGAAATACAAAGACAAATTGAAATTTTA  222
             ||||||||||||||||||||||||||||||||||||||||||||||||||||||||||||
Sbjct  165   TCGATACGTTATATTGTGCAAGCTATAGACTATGAAATACAAAGACAAATTGAAATTTTA  106

Query  223   GAAAGTGGGGAAGAAATAAGTCAAGATACCTTATTGTTTGATGTTGCTTCGGGAAAAACA  282
             ||| ||||||||||||||||||||||||||||||||||||| ||||||||||||||||||
Sbjct  105   GAAGGTGGGGAAGAAATAAGTCAAGATACCTTATTGTTTGACGTTGCTTCGGGAAAAACA  46

Query  283   AAAGTGATGCGAAACAAAGAAGATGCAAGCGACTATAGATACTTC  327
             |||||||||||||||||||||||||||||||||||||||||||||
Sbjct  45    AAAGTGATGCGAAACAAAGAAGATGCAAGCGACTATAGATACTTC  1


> 19292641 length 138 cvg_33.4_tip_0
Length=138

 Score =   255 bits (138),  Expect = 3e-66
 Identities = 138/138 (100%), Gaps = 0/138 (0%)
 Strand=Plus/Minus

Query  164   CGATACGTTATATTGTGCAAGCTATAGACTATGAAATACAAAGACAAATTGAAATTTTAG  223
```

```
           ||||||||||||||||||||||||||||||||||||||||||||||||||||||||||||
Sbjct  138  CGATACGTTATATTGTGCAAGCTATAGACTATGAAATACAAAGACAAATTGAAATTTTAG  79

Query  224  AAAGTGGGGAAGAAATAAGTCAAGATACCTTATTGTTTGATGTTGCTTCGGGAAAAACAA  283
            ||||||||||||||||||||||||||||||||||||||||||||||||||||||||||||
Sbjct  78   AAAGTGGGGAAGAAATAAGTCAAGATACCTTATTGTTTGATGTTGCTTCGGGAAAAACAA  19

Query  284  AAGTGATGCGAAACAAAG  301
            ||||||||||||||||||
Sbjct  18   AAGTGATGCGAAACAAAG  1


> 18969591 length 130 cvg_63.0_tip_0
Length=130

 Score =   230 bits (124),  Expect = 2e-58
 Identities = 128/130 (98%), Gaps = 0/130 (0%)
 Strand=Plus/Plus

Query  64   GATATGGAAAAGGGATCACTTCGTTGTGATGCAAATGTTTCTGTCCGCCTAAAAGGCAGT  123
            ||||||||||||||||||||||||||||||||||||||||||||||||||||||||||||
Sbjct  1    GATATGGAAAAGGGATCACTTCGTTGTGATGCAAATGTTTCTGTCCGCCTAAAAGGCAGT  60

Query  124  AGCGCACTTGGCACTCGTTGTGAGATAAAAAATCTGAACTCGATACGTTATATTGTGCAA  183
            ||| || |||||||||||||||||||||||||||||||||||||||||||||||||||||
Sbjct  61   AGCACATTTGGCACTCGTTGTGAGATAAAAAATCTGAACTCGATACGTTATATTGTGCAA  120

Query  184  GCTATAGACT  193
            ||||||||||
Sbjct  121  GCTATAGACT  130


> 18736645 length 127 cvg_2.0_tip_0
Length=127

 Score =   230 bits (124),  Expect = 2e-58
 Identities = 126/127 (99%), Gaps = 0/127 (0%)
 Strand=Plus/Minus

Query  100  GTTTCTGTCCGCCTAAAAGGCAGTAGCGCACTTGGCACTCGTTGTGAGATAAAAAATCTG  159
            ||||||||||||||||||||||||||||||||||||||||||||||||||||||||||||
Sbjct  127  GTTTCTGTCCGCCTAAAAGGCAGTAGCGCACTTGGCACTCGTTGTGAGATAAAAAATCTG  68

Query  160  AACTCGATACGTTATATTGTGCAAGCTATAGACTATGAAATACAAAGACAAATTGAAATT  219
            ||| ||||||||||||||||||||||||||||||||||||||||||||||||||||||||
Sbjct  67   AACCCGATACGTTATATTGTGCAAGCTATAGACTATGAAATACAAAGACAAATTGAAATT  8

Query  220  TTAGAAA  226
            |||||||
Sbjct  7    TTAGAAA  1


> 18076019 length 127 cvg_63.0_tip_0
Length=127

 Score =   211 bits (114),  Expect = 7e-53
 Identities = 116/117 (99%), Gaps = 0/117 (0%)
 Strand=Plus/Minus

Query  1    GAAGCTGCAGAATGCATGAAAAAATTGAGGCAGATTTTGCGTTACATTGGTTCGTGTGAT  60
            ||||||||||||||||||||||||||||||||||||||||||||||||||||| ||||||
Sbjct  117  GAAGCTGCAGAATGCATGAAAAAATTGAGGCAGATTTTGCGTTACATTGGTTCATGTGAT  58
```

```
Query  61   GGTGATATGGAAAAGGGATCACTTCGTTGTGATGCAAATGTTTCTGTCCGCCTAAAA  117
            |||||||||||||||||||||||||||||||||||||||||||||||||||||||||
Sbjct  57   GGTGATATGGAAAAGGGATCACTTCGTTGTGATGCAAATGTTTCTGTCCGCCTAAAA  1


> 14638785 length 99 cvg_47.0_tip_0
Length=99

 Score =   183 bits (99),  Expect = 1e-44
 Identities = 99/99 (100%), Gaps = 0/99 (0%)
 Strand=Plus/Plus

Query  64   GATATGGAAAAGGGATCACTTCGTTGTGATGCAAATGTTTCTGTCCGCCTAAAAGGCAGT  123
            ||||||||||||||||||||||||||||||||||||||||||||||||||||||||||||
Sbjct  1    GATATGGAAAAGGGATCACTTCGTTGTGATGCAAATGTTTCTGTCCGCCTAAAAGGCAGT  60

Query  124  AGCGCACTTGGCACTCGTTGTGAGATAAAAAATCTGAAC  162
            |||||||||||||||||||||||||||||||||||||||
Sbjct  61   AGCGCACTTGGCACTCGTTGTGAGATAAAAAATCTGAAC  99


> 15078638 length 105 cvg_46.9_tip_0
Length=105

 Score =   176 bits (95),  Expect = 2e-42
 Identities = 95/95 (100%), Gaps = 0/95 (0%)
 Strand=Plus/Minus

Query  1   GAAGCTGCAGAATGCATGAAAAAATTGAGGCAGATTTTGCGTTACATTGGTTCGTGTGAT  60
           ||||||||||||||||||||||||||||||||||||||||||||||||||||||||||||
Sbjct  95  GAAGCTGCAGAATGCATGAAAAAATTGAGGCAGATTTTGCGTTACATTGGTTCGTGTGAT  36

Query  61  GGTGATATGGAAAAGGGATCACTTCGTTGTGATGC  95
           |||||||||||||||||||||||||||||||||||
Sbjct  35  GGTGATATGGAAAAGGGATCACTTCGTTGTGATGC  1


> 14245495 length 95 cvg_63.0_tip_0
Length=95

 Score =   176 bits (95),  Expect = 2e-42
 Identities = 95/95 (100%), Gaps = 0/95 (0%)
 Strand=Plus/Minus

Query  265  GTTGCTTCGGGAAAAACAAAAGTGATGCGAAACAAAGAAGATGCAAGCGACTATAGATAC  324
            ||||||||||||||||||||||||||||||||||||||||||||||||||||||||||||
Sbjct  95   GTTGCTTCGGGAAAAACAAAAGTGATGCGAAACAAAGAAGATGCAAGCGACTATAGATAC  36

Query  325  TTCCCTGAGCCTGATTTATTACCTGTTGAGGTAAG  359
            |||||||||||||||||||||||||||||||||||
Sbjct  35   TTCCCTGAGCCTGATTTATTACCTGTTGAGGTAAG  1


> 21871023 length 258 cvg_1.0_tip_1
Length=258

 Score =   171 bits (92),  Expect = 1e-40
 Identities = 94/95 (99%), Gaps = 0/95 (0%)
 Strand=Plus/Plus

Query  1    GAAGCTGCAGAATGCATGAAAAAATTGAGGCAGATTTTGCGTTACATTGGTTCGTGTGAT  60
            ||||||||||||||||||||||||||||||| ||||||||||||||||||||||||||||
Sbjct  164  GAAGCTGCAGAATGCATGAAAAAATTGAGGCCGATTTTGCGTTACATTGGTTCGTGTGAT  223
```

```
Query  61   GGTGATATGGAAAAGGGATCACTTCGTTGTGATGC  95
            |||||||||||||||||||||||||||||||||||
Sbjct  224  GGTGATATGGAAAAGGGATCACTTCGTTGTGATGC  258


> 13582182 length 89 cvg_32.4_tip_0
Length=89

 Score =   165 bits (89),  Expect = 5e-39
 Identities = 89/89 (100%), Gaps = 0/89 (0%)
 Strand=Plus/Plus

Query  239  TAAGTCAAGATACCTTATTGTTTGATGTTGCTTCGGGAAAAACAAAAGTGATGCGAAACA  298
            ||||||||||||||||||||||||||||||||||||||||||||||||||||||||||||
Sbjct  1    TAAGTCAAGATACCTTATTGTTTGATGTTGCTTCGGGAAAAACAAAAGTGATGCGAAACA  60

Query  299  AAGAAGATGCAAGCGACTATAGATACTTC  327
            |||||||||||||||||||||||||||||
Sbjct  61   AAGAAGATGCAAGCGACTATAGATACTTC  89


> 13402912 length 88 cvg_49.6_tip_0
Length=88

 Score =   163 bits (88),  Expect = 2e-38
 Identities = 88/88 (100%), Gaps = 0/88 (0%)
 Strand=Plus/Minus

Query  100  GTTTCTGTCCGCCTAAAAGGCAGTAGCGCACTTGGCACTCGTTGTGAGATAAAAAATCTG  159
            ||||||||||||||||||||||||||||||||||||||||||||||||||||||||||||
Sbjct  88   GTTTCTGTCCGCCTAAAAGGCAGTAGCGCACTTGGCACTCGTTGTGAGATAAAAAATCTG  29

Query  160  AACTCGATACGTTATATTGTGCAAGCTA  187
            ||||||||||||||||||||||||||||
Sbjct  28   AACTCGATACGTTATATTGTGCAAGCTA  1


> 13056619 length 85 cvg_44.0_tip_0
Length=85

 Score =   158 bits (85),  Expect = 9e-37
 Identities = 85/85 (100%), Gaps = 0/85 (0%)
 Strand=Plus/Plus

Query  33   GATTTTGCGTTACATTGGTTCGTGTGATGGTGATATGGAAAAGGGATCACTTCGTTGTGA  92
            ||||||||||||||||||||||||||||||||||||||||||||||||||||||||||||
Sbjct  1    GATTTTGCGTTACATTGGTTCGTGTGATGGTGATATGGAAAAGGGATCACTTCGTTGTGA  60

Query  93   TGCAAATGTTTCTGTCCGCCTAAAA  117
            |||||||||||||||||||||||||
Sbjct  61   TGCAAATGTTTCTGTCCGCCTAAAA  85


> 13056441 length 85 cvg_63.0_tip_0
Length=85

 Score =   158 bits (85),  Expect = 9e-37
 Identities = 85/85 (100%), Gaps = 0/85 (0%)
 Strand=Plus/Plus

Query  131  TTGGCACTCGTTGTGAGATAAAAAATCTGAACTCGATACGTTATATTGTGCAAGCTATAG  190
            ||||||||||||||||||||||||||||||||||||||||||||||||||||||||||||
```

```
Sbjct  1     TTGGCACTCGTTGTGAGATAAAAAATCTGAACTCGATACGTTATATTGTGCAAGCTATAG  60

Query  191   ACTATGAAATACAAAGACAAATTGA  215
             |||||||||||||||||||||||||
Sbjct  61    ACTATGAAATACAAAGACAAATTGA  85


> 21564999 length 229 cvg_37.0_tip_0
Length=229

 Score =   135 bits (73),  Expect = 4e-30
 Identities = 73/73 (100%), Gaps = 0/73 (0%)
 Strand=Plus/Plus

Query  297   CAAAGAAGATGCAAGCGACTATAGATACTTCCCTGAGCCTGATTTATTACCTGTTGAGGT  356
             ||||||||||||||||||||||||||||||||||||||||||||||||||||||||||||
Sbjct  1     CAAAGAAGATGCAAGCGACTATAGATACTTCCCTGAGCCTGATTTATTACCTGTTGAGGT  60

Query  357   AAGGCAGGATAAA  369
             |||||||||||||
Sbjct  61    AAGGCAGGATAAA  73


> 10262355 length 73 cvg_63.0_tip_0
Length=73

 Score =   135 bits (73),  Expect = 4e-30
 Identities = 73/73 (100%), Gaps = 0/73 (0%)
 Strand=Plus/Plus

Query  153   AAATCTGAACTCGATACGTTATATTGTGCAAGCTATAGACTATGAAATACAAAGACAAAT  212
             ||||||||||||||||||||||||||||||||||||||||||||||||||||||||||||
Sbjct  1     AAATCTGAACTCGATACGTTATATTGTGCAAGCTATAGACTATGAAATACAAAGACAAAT  60

Query  213   TGAAATTTTAGAA  225
             |||||||||||||
Sbjct  61    TGAAATTTTAGAA  73


> 10213031 length 72 cvg_63.0_tip_0
Length=72

 Score =   134 bits (72),  Expect = 2e-29
 Identities = 72/72 (100%), Gaps = 0/72 (0%)
 Strand=Plus/Plus

Query  55    TGTGATGGTGATATGGAAAAGGGATCACTTCGTTGTGATGCAAATGTTTCTGTCCGCCTA  114
             ||||||||||||||||||||||||||||||||||||||||||||||||||||||||||||
Sbjct  1     TGTGATGGTGATATGGAAAAGGGATCACTTCGTTGTGATGCAAATGTTTCTGTCCGCCTA  60

Query  115   AAAGGCAGTAGC  126
             ||||||||||||
Sbjct  61    AAAGGCAGTAGC  72


> 8722663 length 69 cvg_47.0_tip_0
Length=69

 Score =   128 bits (69),  Expect = 7e-28
 Identities = 69/69 (100%), Gaps = 0/69 (0%)
 Strand=Plus/Minus

Query  125   GCGCACTTGGCACTCGTTGTGAGATAAAAAATCTGAACTCGATACGTTATATTGTGCAAG  184
```

```
            ||||||||||||||||||||||||||||||||||||||||||||||||||||||||||||
Sbjct  69   GCGCACTTGGCACTCGTTGTGAGATAAAAAATCTGAACTCGATACGTTATATTGTGCAAG  10

Query  185  CTATAGACT  193
            |||||||||
Sbjct  9    CTATAGACT  1


> 20838530 length 194 cvg_62.4_tip_0
Length=194

 Score =   124 bits (67),  Expect = 9e-27
 Identities = 71/73 (97%), Gaps = 0/73 (0%)
 Strand=Plus/Plus

Query  297  CAAAGAAGATGCAAGCGACTATAGATACTTCCCTGAGCCTGATTTATTACCTGTTGAGGT  356
            ||||||||||||||||||||||||||||||||||||||||||||||||||||||||||||
Sbjct  1    CAAAGAAGATGCAAGCGACTATAGATACTTCCCTGAGCCTGATTTATTACCTGTTGAGGT  60

Query  357  AAGGCAGGATAAA  369
            ||| ||||| |||
Sbjct  61   AAGCCAGGAGAAA  73


> 3629341 length 64 cvg_0.0_tip_0
Length=64

 Score =   119 bits (64),  Expect = 4e-25
 Identities = 64/64 (100%), Gaps = 0/64 (0%)
 Strand=Plus/Plus

Query  163  TCGATACGTTATATTGTGCAAGCTATAGACTATGAAATACAAAGACAAATTGAAATTTTA  222
            ||||||||||||||||||||||||||||||||||||||||||||||||||||||||||||
Sbjct  1    TCGATACGTTATATTGTGCAAGCTATAGACTATGAAATACAAAGACAAATTGAAATTTTA  60

Query  223  GAAA  226
            ||||
Sbjct  61   GAAA  64



Lambda      K        H
    1.33    0.621     1.12

Gapped
Lambda      K        H
    1.28    0.460    0.850

Effective search space used: 349465373422
```
