# Supplementary material for: Tracing the history and ecological context of Wolbachia double infection in a specialist host (Urophora cardui)—parasitoid (Eurytoma serratulae) system
Source: Ecol Evol. 2017 Jan 17;7(3):986–96. doi: 10.1002/ece3.2713 (PMC5288247; doi:10.1002/ece3.2713)
Supplement: Supplementary file 4 [file ECE3-7-986-s004.pdf]

Appendix S4

Johannesen, J.: Tracing the history and ecological context of *Wolbachia* double infection in a specialist host (*Urophora cardui*) – parasitoid (*Eurytoma serratulae*) system

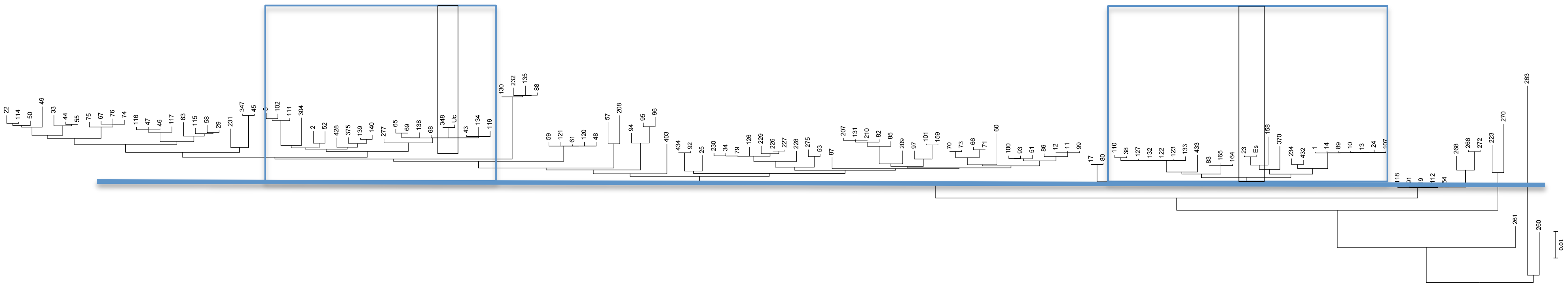

Neighbor-Joining tree (K2P model) of 131 *Wolbachia* supergroup A strains based on full MLST. Boxes in blue show the position of subtrees with Ucar_A1 (Uc) and Eser_A (Es) presented in Fig. 3. Black boxes show the positions of Ucar_A1 and Eser_A within subtrees. The tree was calculated from 2071 positions in the final data set (gaps deleted). Total branch length of optimal tree = 0.67672.
