# Supplementary material for: Tracing the history and ecological context of Wolbachia double infection in a specialist host (Urophora cardui)—parasitoid (Eurytoma serratulae) system
Source: Ecol Evol. 2017 Jan 17;7(3):986–96. doi: 10.1002/ece3.2713 (PMC5288247; doi:10.1002/ece3.2713)

Appendix S5. **Johannesen, J.: Tracing the history and ecological context of *Wolbachia* double infection in a specialist host (*Urophora cardui*) – parasitoid (*Eurytoma serratulae*) system**

Regression of double infection (DI) on *E. serratulae* parasitisation rates in *U. cardui* populations. The analysis was performed on 44 populations for which both parameters were available.

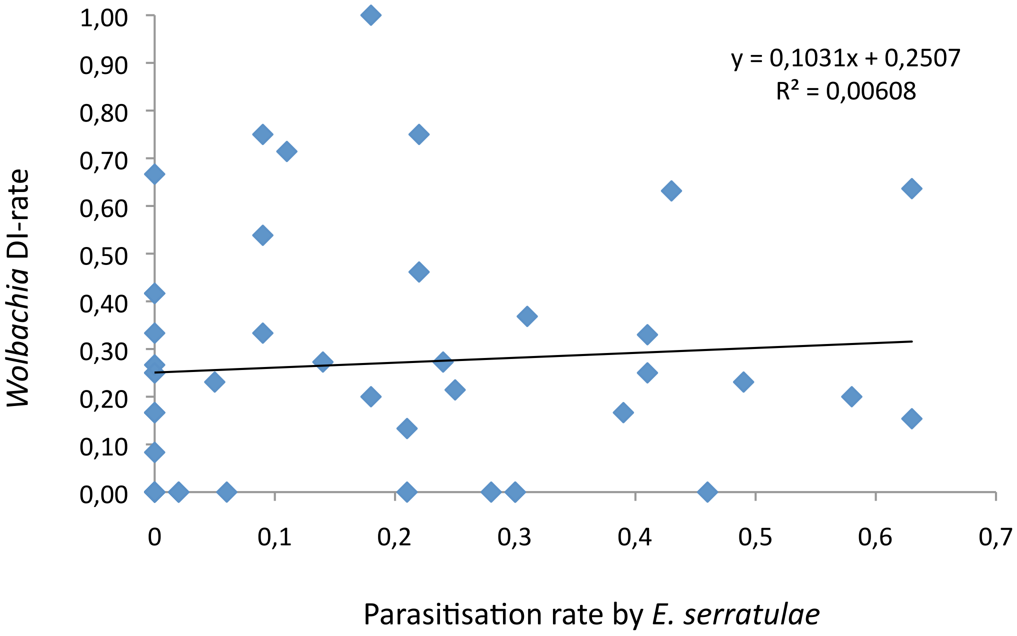

Supplement: Supplementary file 5 [file ECE3-7-986-s005.pdf]
